# Fronts divide diazotroph communities in the Southern Indian Ocean

**DOI:** 10.1093/femsec/fiae095

**Published:** 2024-07-11

**Authors:** Subhadeep Chowdhury, Hugo Berthelot, Corentin Baudet, David González-Santana, Christian Furbo Reeder, Stéphane L'Helguen, Jean-François Maguer, Carolin R Löscher, Arvind Singh, Stéphane Blain, Nicolas Cassar, Sophie Bonnet, Hélène Planquette, Mar Benavides

**Affiliations:** Aix Marseille Université, CNRS, Université de Toulon, IRD, OSU Pythéas, Mediterranean Institute of Oceanography (MIO), UM 110, 13288 Marseille, France; Turing Center for Living Systems, Aix-Marseille University, Marseille, France; IFREMER, DYNECO, Pelagos Laboratory, Plouzané, France; Laboratoire des Sciences de l'Environnement Marin, IUEM, Université de Brest-UMR 6539 CNRS/UBO/IRD, Technopole Brest-Iroise, 29280 Plouzané, France; Laboratoire des Sciences de l'Environnement Marin, IUEM, Université de Brest-UMR 6539 CNRS/UBO/IRD, Technopole Brest-Iroise, 29280 Plouzané, France; Univ Brest, CNRS, IRD, IFREMER, LEMAR, F-29280 Plouzané, France; Instituto de Oceanografía y Cambio Global, IOCAG, Universidad de Las Palmas de Gran Canaria, Las Palmas de Gran Canaria, Spain; Aix Marseille Université, CNRS, Université de Toulon, IRD, OSU Pythéas, Mediterranean Institute of Oceanography (MIO), UM 110, 13288 Marseille, France; Turing Center for Living Systems, Aix-Marseille University, Marseille, France; Laboratoire des Sciences de l'Environnement Marin, IUEM, Université de Brest-UMR 6539 CNRS/UBO/IRD, Technopole Brest-Iroise, 29280 Plouzané, France; Laboratoire des Sciences de l'Environnement Marin, IUEM, Université de Brest-UMR 6539 CNRS/UBO/IRD, Technopole Brest-Iroise, 29280 Plouzané, France; Nordcee, Department of Biology, University of Southern Denmark, Odense 5230, Denmark; Geosciences Division, Physical Research Laboratory, Ahmedabad 380009, India; Sorbonne Université, CNRS, Laboratoire d'Océanographie Microbienne (LOMIC), Observatoire Océanologique de Banyuls, 66650 Banyuls/mer, France; Laboratoire des Sciences de l'Environnement Marin, IUEM, Université de Brest-UMR 6539 CNRS/UBO/IRD, Technopole Brest-Iroise, 29280 Plouzané, France; Division of Earth and Climate Sciences, Nicholas School of the Environment, Duke University, Durham, NC 27708, United States; Aix Marseille Université, CNRS, Université de Toulon, IRD, OSU Pythéas, Mediterranean Institute of Oceanography (MIO), UM 110, 13288 Marseille, France; Laboratoire des Sciences de l'Environnement Marin, IUEM, Université de Brest-UMR 6539 CNRS/UBO/IRD, Technopole Brest-Iroise, 29280 Plouzané, France; Aix Marseille Université, CNRS, Université de Toulon, IRD, OSU Pythéas, Mediterranean Institute of Oceanography (MIO), UM 110, 13288 Marseille, France; Turing Center for Living Systems, Aix-Marseille University, Marseille, France

**Keywords:** fronts, HNLC, N_2_ fixation, noncyanobacterial diazotrophs, subtropical gyre, trace metals

## Abstract

Dinitrogen (N_2_) fixation represents a key source of reactive nitrogen in marine ecosystems. While the process has been rather well-explored in low latitudes of the Atlantic and Pacific Oceans, other higher latitude regions and particularly the Indian Ocean have been chronically overlooked. Here, we characterize N_2_ fixation and diazotroph community composition across nutrient and trace metals gradients spanning the multifrontal system separating the oligotrophic waters of the Indian Ocean subtropical gyre from the high nutrient low chlorophyll waters of the Southern Ocean. We found a sharp contrasting distribution of diazotroph groups across the frontal system. Notably, cyanobacterial diazotrophs dominated north of fronts, driving high N_2_ fixation rates (up to 13.96 nmol N l^−1^ d^−1^) with notable peaks near the South African coast. South of the fronts non-cyanobacterial diazotrophs prevailed without significant N_2_ fixation activity being detected. Our results provide new crucial insights into high latitude diazotrophy in the Indian Ocean, which should contribute to improved climate model parameterization and enhanced constraints on global net primary productivity projections.

## Teaser

Fronts dividing the Indian Ocean and the Southern Ocean imprint a biogeochemical divide structuring diazotroph communities.

## Introduction

Prokaryotes called “diazotrophs” fix dinitrogen (N_2_) into ammonium, sustaining primary productivity and carbon export in the ocean (Karl et al. 1997, Zehr and Capone 2020, Bonnet et al. 2023). Although cyanobacteria are conventionally regarded as the primary contributors to marine N_2_ fixation, non-cyanobacterial diazotrophs (NCDs) are ubiquitously distributed in marine ecosystems (Turk-Kubo et al. 2022). The activity of diazotrophs is importantly controlled by temperature (Sohm et al. 2011), as well as the availability of phosphorus and iron (Fe) (Mills et al. 2004). N_2_ fixation can be inhibited by reactive nitrogen compounds such as ammonium or nitrate (LaRoche and Breitbarth 2005). Under such constraints, N_2_ fixation has long been assumed to be restricted to warm low-latitude nitrate-poor open ocean regions, provided sufficient phosphorus and Fe are available (Zehr and Capone 2020). However, recent studies reported significant N_2_ fixation activity in cold an nutrient-rich regions, including temperate (Raes et al. 2020), and polar waters (von Friesen and Riemann 2020).

The Southern Indian Ocean (SIO) region comprises the Indian Ocean (IO) subtropical gyre and the Indian sector of the Southern Ocean. The IO subtropical gyre is characterized by low dissolved nitrogen-to-phosphorus (N:P) ratios (∼4:1) in the photic zone (Baer et al. 2019), conditions believed to favour diazotrophy (Knapp 2012). However, low Fe availability in the IO subtropical gyre may hinder N_2_ fixation and intensify nitrogen limitation (Grand et al. 2015). Recurring massive blooms of *Trichodesmium*, a prominent diazotrophic cyanobacterium, and frequent occurrence of diatom–diazotroph associations are observed at the southern tip of Madagascar (Poulton et al. 2009). These observations point towards an important contribution of diazotrophs to nitrogen fluxes in the IO’s subtropical gyre (Poulton et al. 2009, Metzl et al. 2022).

The IO subtropical gyre is separated from the Indian sector of the Southern Ocean by a complex succession of quasi-zonal frontal structures, including the subtropical front (STF), subantarctic Front (SAF), and polar front (PF) (Kostianoy et al. 2004, Chapman et al. 2020). While fronts are usually considered as barriers, recent studies have shown that front-associated filaments facilitate sub/mesoscale transfer of seawater properties and communities between the Southern Ocean and the IO subtropical gyre across the fronts (Read et al. 2000, Hörstmann et al. 2021). Fronts can enhance vertical fluxes of nutrients (D’Asaro et al. 2011), resulting in high primary productivity, and typically distinct microbial communities as compared to the surrounding water masses (Baltar et al. 2016, Hörstmann et al. 2021). While some studies have investigated diazotrophy across frontal systems (Fong et al. 2008, Benavides et al. 2011, 2021, Riou et al. 2016, Shiozaki et al. 2018, Jiang et al. 2019), their role in structuring diazotroph communities and controlling N_2_ fixation inputs is largely unknown (Benavides and Robidart 2020).

The Southern Ocean is rich in macronutrients but low in productivity due to the lack of Fe, for which it is known as a “high nutrient low chlorophyll” (HNLC) region (Venables and Moore 2010). Diazotrophs rely heavily on Fe, a vital component of the nitrogenase enzyme (Berman-Frank et al. 2001). The lack of Fe together with the cold-water conditions and high nitrate concentrations of the Southern Ocean IO sector are thus expected to suppress diazotrophy. However, a range of processes provides intermittent sources of Fe in this region, including atmospheric deposition, frontal jet interactions with bathymetry (Blain et al. 2007, Klocker 2018), and Fe from hydrothermal origin (Ardyna et al. 2019, Schine et al. 2021), known to prompt phytoplankton blooms and possibly diazotrophic activity as well. In addition to Fe, bioactive trace metals such as manganese (Mn), cobalt (Co), nickel (Ni), copper (Cu), and zinc (Zn) are essential enzyme cofactors, controlling the growth of primary producers in the open ocean (Saito et al. 2008, Sunda 2012, Balaguer et al. 2023). However, the effects of trace metals, excluding Fe, on diazotrophs are not well understood.

An intercomparison of the latest generation of Earth system models shows that the IO is one of the main ocean basins adding uncertainty to global net primary productivity projections (Tagliabue et al. 2021). Hence, increasing N_2_ fixation measurements and its controlling mechanisms in the IO is crucial for improving the predictability of net primary productivity and understanding the ocean’s future role as a climate change regulator. Seeking to improve the coverage of diazotrophy studies in the SIO, here we investigate N_2_ fixation rates and the diazotroph community composition and abundance along a transect spanning from the oligotrophic IO subtropical gyre to the HNLC waters of the Southern Ocean. This study provides valuable data to improve our understanding of high latitude diazotrophy and nitrogen budgets in the IO.

## Materials and methods

### Sample collection and environmental parameters

**Figure 1.**
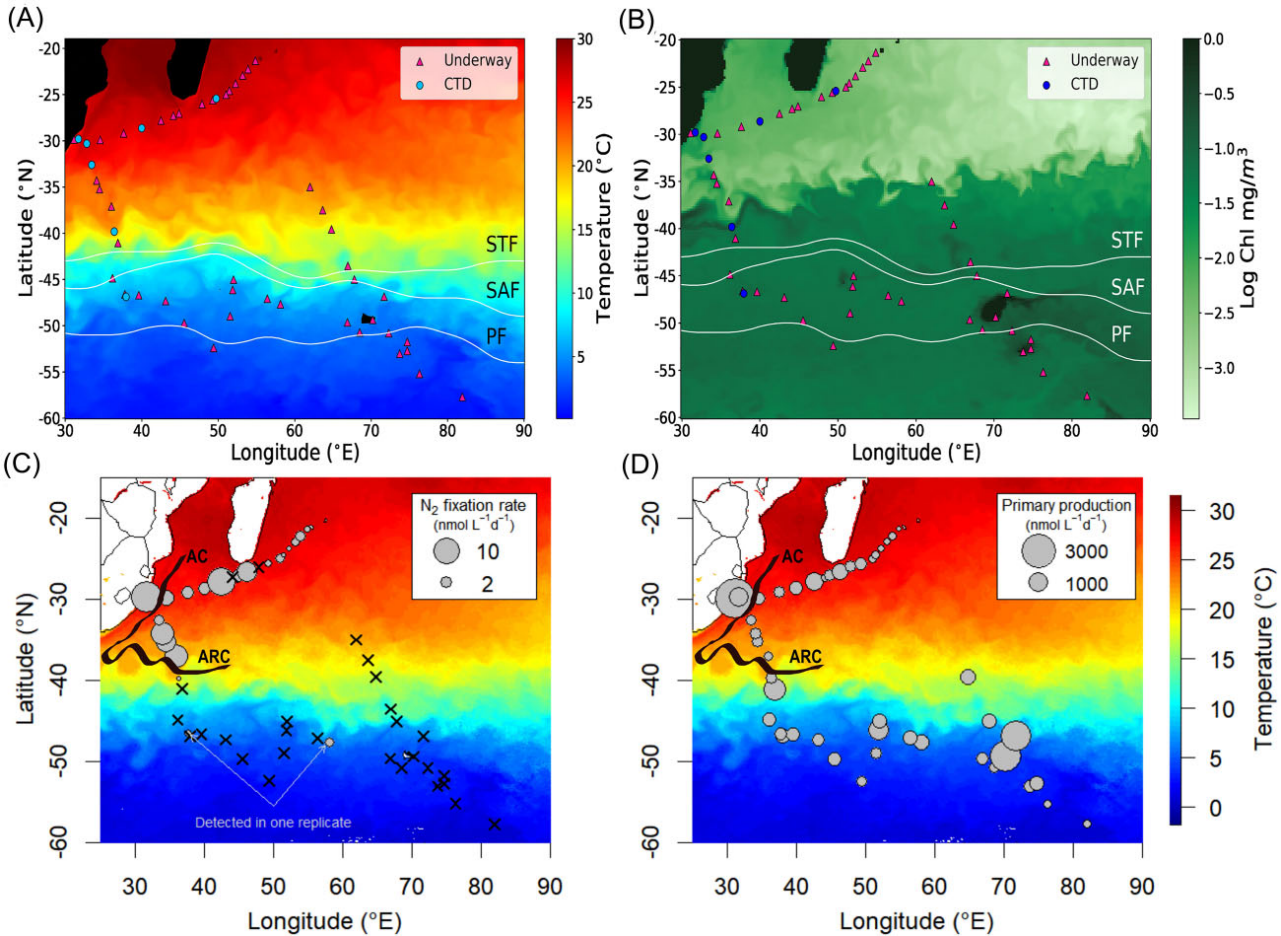
CTD profile and underway stations sampled during the cruise overlaid on (A) sea surface temperature (SST) and Chlorophyll-*a* composite of L3M 4 km product retrieved from the Copernicus marine service for the cruise period (13 January 2021 to 4 March 2021). STF = subtropical front, SAF = subantarctic front, and PF = polar front. (C) N_2_ fixation (nmol N l^−1^ d^−1^) and (D) primary production (nmol C l^−1^ d^−1^) rates measured from surface samples (5 m) along the cruise transect. Nondetectable rates are depicted with crosses. AC = Agulhas current and ARC = Agulhas return current.

This study was conducted from 13 January to 4 March 2021 (austral summer) onboard the R/V *Marion Dufresne* II as part of the French SWINGS (GEOTRACES GS02) cruise (doi: 10.17600/18001925) (Fig. 1). Surface seawater was collected at eight stations using Niskin bottles mounted on a conductivity-temperature-depth (CTD) rosette package and at 43 other stations using the underway sampling system (Fig. 1). Underway sampling consisted of a water intake at the base of the ship hull through the moonpool of the ship and a PTFE pump and tubing minimizing trace metal contaminations. Sea surface temperature (SST) and salinity data were retrieved from the CTD sensors. Chlorophyll *a* concentrations were retrieved from Aqua MODIS Satellite data (from 13 January to 4 March 2021, L3M 4 km product). Samples for dissolved inorganic nutrients and trace metals were collected from CTD casts using a trace metal clean polyurethane powder-coated aluminum frame rosette equipped with 24 12-l, externally closing, Teflon-lined, GO-FLO bottles (General Oceanics) and attached to a Kevlar® line, according to the GEOTRACES guidelines (Cutter et al. 2017). Seawater samples for dissolved inorganic nutrients analysis (nitrate, nitrite, phosphate, and silicic acid) were prefiltered through 0.45-µm acetate cellulose filters, poisoned with HgCl_2_ (4 g l^−1^) and stored in the dark at room temperature until analysis using a Skalar segmented flow autoanalyzer (Blain et al. 2015). Detection limits for the nitrate, nitrite, phosphate, and silicic acid were 0.15 µmol l^−1^, 0.01 µmol l^−1^, 0.02 µmol l^−1^, and 0.07 µmol l^−1^, respectively.

Samples for dissolved trace metals measurements (cobalt—Co-, iron—Fe-, manganese—Mn-, nickel—Ni-, lead—Pb-, and zinc—Zn-) were obtained from discrete stations (Fig. 1) following the methods outlined in the Geotraces Cookbook (https://geotracesold.sedoo.fr/images/Cookbook.pdf). Analyses were carried out within 12 months after collection on an SF-HR-ICP-MS Element XR instrument (Thermo Fisher, Bremen, Germany), at Pôle Spectrométrie Océan (IFREMER, France). The spectrometer was coupled to an ESI seaFAST-pico® introduction system and run with a method analytically similar to that of Lagerström et al. (2013).

Dissolved nutrients and trace metal concentration data are used here for statistical purposes, while a full description of their variability during the SWINGS cruise will be provided in dedicated papers (e.g. Baudet et al., submitted).

### N_2_ fixation and primary production rates

Samples for the measurement of primary production and N_2_ fixation rates were collected from the underway outlet as a set of four 2.3-l acid-washed polycarbonate bottles. Three bottles were spiked with 2.3 ml of a ^13^C-labelled bicarbonate solution (NaH^13^CO_2_; >98%, Sigma Aldrich, 10 atom% final ^13^C abundance) and 2.3 ml of ^15^N_2_ (^15^ N isotopic abundance of 99.7%, Eurisotop, Saclay, France) using the bubble method to maximize the final ^15^N isotopic abundance and improve the detection limit. Bottles were inverted at least 60 times before incubation to ensure rapid isotopic equilibrium. The fourth bottle was left unamended and used as a control. All the bottles were incubated for 24 h in an on-deck incubator with circulating surface water and a blue filter reproducing the light at the sampling depth. Temperature changes between the start and the end of the incubation were limited to 1.2°C on average. Following incubations, 12 ml of water were syphoned from the bottles in Exetainer tubes and poisoned with HgCl_2_ for ^15^N_2_ isotopic abundance analyses. The remaining bottle content was filtered on combusted (450°C, 4 h) 25-mm diameter glass fibre filters (Whatman, London, UK). Filters were stored at −20°C before being dried for downstream analysis (24 h, 60°C). The ^15^N_2_ isotopic abundance in water was measured within 6 months using a membrane inlet mass spectrometer according to Kana et al. (1994). The particulate carbon and nitrogen isotopic enrichment (^13^C and ^15^N) was measured on an elemental analyzer coupled to an isotope ratio mass spectrometer (Deltaplus, Thermo Finnigan). N_2_ fixation rates were calculated according to Montoya et al. (1996). Minimum quantifiable rates were also calculated based on error propagation of the replicates as proposed by White et al. (2020).

Single-cell N_2_ fixation rates were measured by nanoscale secondary ion mass spectrometry (nanoSIMS) as described in Bonnet et al. (2016) at station 857 (north of the fronts) targeting *Crocosphaera*-like cells and *Trichodesmium*, and at four stations south of the fronts (stations 872, 876, 887, and 893) targeting putative NCDs. Putative NCDs were not measured north of the fronts (where cyanobacterial N_2_ fixation rates were expected to be high), to avoid false positives due to the transfer of enriched ^15^N biomass from cyanobacterial diazotrophs to heterotrophic bacteria (Bonnet et al. 2016). Briefly, at station 857 cells were deposited on a polycarbonate filter (0.2 µm pore size) following ^15^N_2_ incubation. *Trichodesmium* filament and *Crocosphaera*-like cells were mapped by epifluorescence before nanoSIMS analyses. At stations 872, 876, 887, and 893, incubated cells were laid on a polycarbonate filter (0.2 µm pore size) and resuspended in 4.5 ml freshly produced filtered (0.2 µm pore size) seawater, fixed with 1% paraformaldehyde and flash frozen in liquid nitrogen. Cells were then stored at −80°C. Back in the laboratory, samples were thawed and cells were labelled with SYBR green I DNA dye (Molecular Probes, final concentration 0.05%). Heterotrophic bacteria were gated according to Marie et al. (1999) and sorted using a BD FACSAria™ Fusion cell sorter. Sorted heterotrophic bacteria were directly deposited on a polycarbonate membrane on the sorter outlet as described by Bonnet et al. (2016). In total, 50 cyanobacterial diazotrophs were analyzed, and 2,535 heterotrophic bacteria. Incubation time was 24 h for station 857 and between 48 and 168 h, long enough to increase the likelihood of detecting positively enriched NCDs. Cells were considered as significantly ^15^N enriched when the number of ^15^N ions counted exceeded the number of ^15^N ions assuming a natural abundance (0.366 atom%) plus three times the standard deviation of the Poisson distribution modelled for each cell (see Berthelot et al. (2019) for more details).

### DNA sampling, extraction, diazotroph abundance, and *nifH/nifD* gene sequencing

Nucleic acids were sampled from 51 stations, 2-l seawater samples filtered through 0.2-µm polycarbonate filters, and stored at −80°C until analyzed. DNA was extracted using the DNeasy Plant Mini Kit (Qiagen, CA, USA) and quantified by the Picogreen method using a VarioSkan spectrofluorometer (Thermo Fisher Scientific, Massachusetts, USA). For DNA extraction, we used additional modifications including freeze–thaw with liquid nitrogen, bead beating, and Proteinase-K steps before the kit purification and elution to 70 µl in RNase-free water, as previously described (Moisander et al. 2008). The abundance of *nif*H genes was quantified using TaqMan-specific quantitative PCR (qPCR) assays targeting six diazotroph groups including *Trichodesmium*, UCYN-A1, UCYN-A2, UCYN-B, Gamma-A, and Gamma-4, using published primer–probe sets (see Supplemental files).

The *nif*H gene was amplified using degenerated oligonucleotide primers (*nif*H1–2–3–4) (Zehr and McReynolds 1989), to produce a final amplicon length of 359 base pairs (bp). In diazotroph community studies, *nifH* is frequently employed as a biomarker gene to identify diazotrophs and their community composition (Frank et al. 2016, Gaby et al. 2018). However, to perform N_2_ fixation diazotrophs require a minimum set of genes (*nif*HDKENB; Dos Santos et al. 2012). Up to 20% of *nif*H-harbouring genomes showed the absence of the genes *nif*D and *nif*K, and this genomic configuration is referred to as pseudo *nif*H (Mise et al. 2021). Pseudo *nif*H has been found in various common diazotroph groups, including Clostridia and methanogens (Mise et al. 2021). A recent study advocated rethinking using *nif*H as the primary biomarker for N_2_ fixing microbes and proposed to consider the *nif*D and *nif*K genes instead (Mise et al. 2021). Here, we used a dual gene amplicon approach (*nif*H and *nif*D genes) to comprehensively explore diazotroph community composition. To amplify *nif*D genes, nested PCRs were performed to produce a final amplicon length of 512 bp (McRose et al. 2017). Amplicons were checked using a 1.2% agarose gel stained with ethidium bromide, and images were captured using a UV transilluminator. *nif*H and *nif*D PCR fragments were purified by using the MP Biomedicals Geneclean Turbo Kit (Fisher Scientific, Massachusetts, USA). Gel-purified PCR products were shipped to Macrogen, Inc. (Amsterdam, Netherlands) for the clone library preparation, adapter ligation, and Illumina MiSeq 2 × 300 bp paired-end sequencing.

### Bioinformatics

Demultiplexed raw Illumina sequence reads were received from the sequencing facility and primer and adapter sequences removed. Sequences were filtered, trimmed, and processed using the DADA2 (V. 1.16) pipeline with default settings (Callahan et al. 2016). After reviewing the quality profiles, sequences with a quality score >30% were kept for downstream processing. Upon concatenating all the processed merged reads from the forward and reverse sequences, only sequences exceeding a minimum length of 300 bp were retained for subsequent downstream analyses. Chimeras were removed to get rid of the artefact sequences created by two or more biological sequences that were wrongly linked together. Post chimera removal, amplicon sequence variants (ASVs) with a length greater than 300 bp are selected for further analysis, including ASV table generation and taxonomic profiling. To exclude the potential non-*nif*H reads, we applied the specific following segments of the NifMAP pipeline v.1.0 (Angel et al. 2018). (a) The filtration was conducted utilizing the Hidden–Markov Model (HMM) “hmm_nuc_1160_*nif*H.hmm” through the application of hmmsearch in HMMER version 3.1b2 (http://hmmer.org/). (b) Amino acid translation, adjusting for potential frameshifts, was done using Framebot (Wang et al. 2013) against a *nif*H reference set. (c) Detection of *nif*H homologs (*bchX, chlL, bchL*, and *parA*) was done employing the HMM “*nif*H_chlL_bchX.hmm” through the utilization of hmmscan in HMMER. (d) Filtered ASVs were annotated into clusters using the CART model (Frank et al. 2016). ASVs that successfully underwent the NifMAP pipeline were retained for further downstream analysis. The taxonomy of the *nif*H ASVs was assigned by the *nif*H database v2.0.5 (Moynihan and Reeder 2023).

We used the *nifD* database developed by Furbo Reeder et al. (2023) (doi:10.5281/zenodo.10124357). For the development of the *nif*D database, ARBitrator (Heller et al. 2014) (version 14 April 2022) were used for retrieval of *nif*D sequences from GenBank matching a given set of reference sequences. Reference sequences were verified as true *nif*D using CD-search tool (CD-HIT) (Lu et al. 2020) to check if they contained the conserved protein domain (cd01976). Settings for Arbitrator were as follows: quality and superiority thresholds were set to 8.1 and 0.1, as in Heller et al. (2014). As a model for conserved domains (cd) models, cd01976 was used as a positive indicator while cd01967 was used as an indicator for an uninformative domain. *nif*D sequences were stored as EMBL records, which were imported into ARB (Ludwig et al. 2004) to create a *nif*D ARB database. Finally, the *nif*D ARB database was exported in XML format. Sequence ID, taxa, fasta sequence, and accession number, were extracted using Moynihan and Reeder (2023) R script. In total, we obtained 2438 *nif*D sequences. ASVs generated by *nif*D sequencing were assigned by our newly formed *nif*D database nifD_DB_v1.1.0 (https://github.com/OceanBridges/nifDdada2).

The top 100 ASVs from *nif*H and *nif*D were queried against the NCBI nucleotide databases to find related reference sequences. Sequences were aligned using the MUSCLE algorithm (Edgar 2004) in MEGA X (Kumar et al. 2018). To ascertain the optimal nucleotide substitution model and assess the need for gamma correction or estimation of invariable sites, the aligned *nif*H and *nif*D sequences underwent a model test using raxmlGUI 2.0 (Edler et al. 2021). Following the model selection process, determined by the Akaike Information Criterion, a maximum-likelihood phylogenetic tree was constructed with 1000 bootstrap replicates using raxmlGUI 2.0 (Edler et al. 2021). The trees were subsequently visualized and annotated using iTOL (https://itol.embl.de/) (Letunic and Bork 2021).

### Statistical analyses

Since temperature is a major factor influencing the distributions of diazotrophs (Stal 2009, Sohm et al. 2011), we employed Ward’s method to cluster stations based on SST. This approach aimed to enhance our understanding of diazotroph distribution patterns and their temperature preferences. Data analysis was conducted using R (v4.2.0), and data visualization employed ggplot2 (v3.4.0) (Wickham 2009). Dendrogram hierarchical clustering with Ward’s method (Ward Jr. 1963) was applied to cluster the sampled stations based on the SST (Fig. S1, Supplemental files). Pearson correlations were used to assess pairwise associations between inorganic nutrients, physical parameters, and trace metal concentrations (collectively defined here as “environmental variables”), N_2_ fixation rates, and diazotroph *nif*H gene counts, using the rcorr function from the corrr package (v0.6.0) (Makowski et al. 2020). Differences in diazotroph community composition as assessed by *nif*H and *nif*D amplicon sequencing were investigated by nonmetric dimensional scaling analysis using the metaMDS from the vegan package The effect of environmental variables on diazotroph community composition and abundance was examined using redundancy analysis from the vegan package (Oksanen et al. 2019). Temperature preferences of predominant diazotroph groups are depicted using ternary plots (https://www.ternaryplot.com/).

## Results

### Description of the study area and environmental settings

Strong gradients in SST, dissolved inorganic nutrients, trace metals, and Chlorophyll-*a* concentrations were observed across the fronts separating the IO subtropical gyre and Southern Ocean waters. Among these variables, SST was the best discriminator to group stations along the cruise transect, as revealed by a dendrogram analysis distinguishing three distinct SST clusters including SST >25°C, SST between 10°C and 25°C, and SST <10°C (Fig. S1, Supplemental files).

Nitrate plus nitrite, and phosphate concentrations were below the analytical detection limit north of the fronts, and were 10–20 µM and up to 2 µM, respectively, south of the STF (Fig. S2, Supplemental files). Silicic acid showed a more patchy distribution, with generally low values (<5 µM) but peak concentrations at stations located close to islands (>10 and up to 20 µM; Fig. S2, Supplemental files). The distribution of dissolved trace metals (dPb, dMn, dCo, dFe, dNi, and dZn) varied across the fronts (Fig. S3, Supplemental files). North of the fronts, higher concentrations of dPb (0.02–0.04 nM) and dMn (0.47–1.67 nM) were observed (Fig. S3, Supplemental files). In contrast, south of the fronts, increased levels of dCo (0–0.06 nM), dFe (0.03–5.08 nM), dNi (3.11–6.28 nM), and dZn (0.42–4.38 nM) were detected (Fig. S3, Supplemental files). High chlorophyll-*a* concentration patches were detected south of the fronts, particularly near the French Southern and Antarctic lands (Amsterdam, Crozet, Kerguelen Islands) in the Southern Ocean (Fig. 1B).

### N_2_ fixation and primary production rates

**Figure 2.**
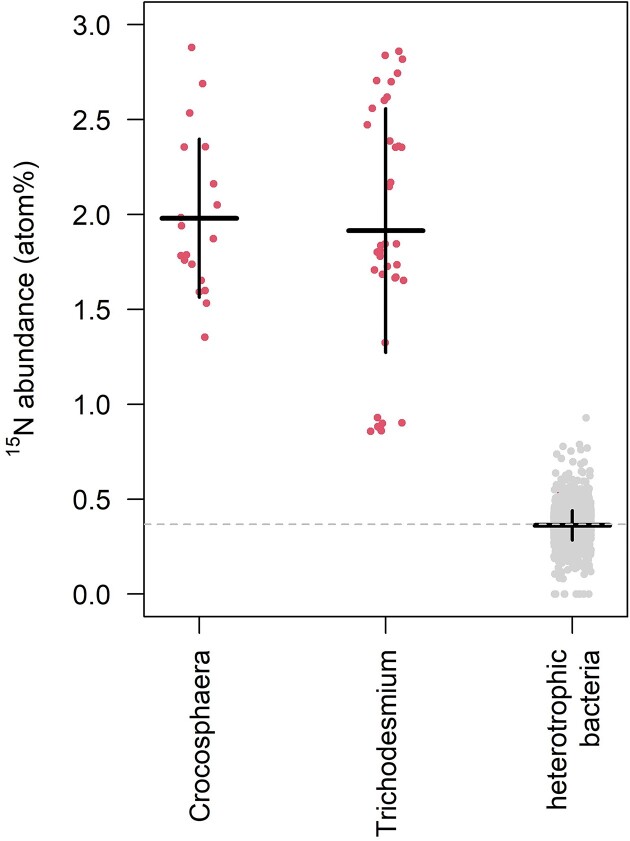
Whisker plot of ^15^N cellular fractional abundance (atom%) for each group analyzed. Each dot represents a single analyzed cell. Gray dots denote cells with rates not significantly different from zero. Black lines denote mean ^15^N fractional abundance and standard deviations (horizontal plain and vertical dashed, respectively). ^15^N enrichments of heterotrophic bacteria were significantly different from *Crocosphaera* and *Trichodesmium* (Wilkoxon test, *P* < .001), but not *Crocosphaera* and*Trichodesmium*  ^15^N enrichments were not significantly different from each other (Wilkoxon test, *P* = .92).

N_2_ fixation rates ranged from below the detection limit to 13.96 nmol N l^−1^ d^−1^ north of the fronts, and from below the detection limit to 1.23 nmol N l^−1^ d^−1^ south of the fronts (Fig. 1C). N_2_ fixation rates peaked south of Madagascar and at stations near the South African coast, influenced by the Agulhas current (Fig. 1C). N_2_ fixation rates were otherwise below the detection limit at most stations south of the fronts as well as at stations on the eastern part of the transect (∼70°E, Fig. 1C). Primary production rates ranged from 93 to 4044 nmol C l^−1^ d^−1^, and 182 to 2456 nmol C l^−1^ d^−1^ north and south of the fronts, respectively (Fig. 2B). These rates were more unevenly distributed than N_2_ fixation rates, with the highest rates detected at stations 866 (4044 nmol C l^−1^ d^−1^, near the South African coast, north of the fronts) and 895 (2456 nmol C l^−1^ d^−1^, near Kerguelen Islands, south of the fronts) (Fig. 1D). While both *Trichodesmium* and *Crocosphaera* cells were found to be significantly enriched in ^15^N north of the fronts, combining flow cytometry and nanoSIMS we screened more than 2,500 heterotrophic bacteria south of the fronts and we could not find any significant enrichment (Fig. 2).

### Diazotroph abundance

**Figure 3.**
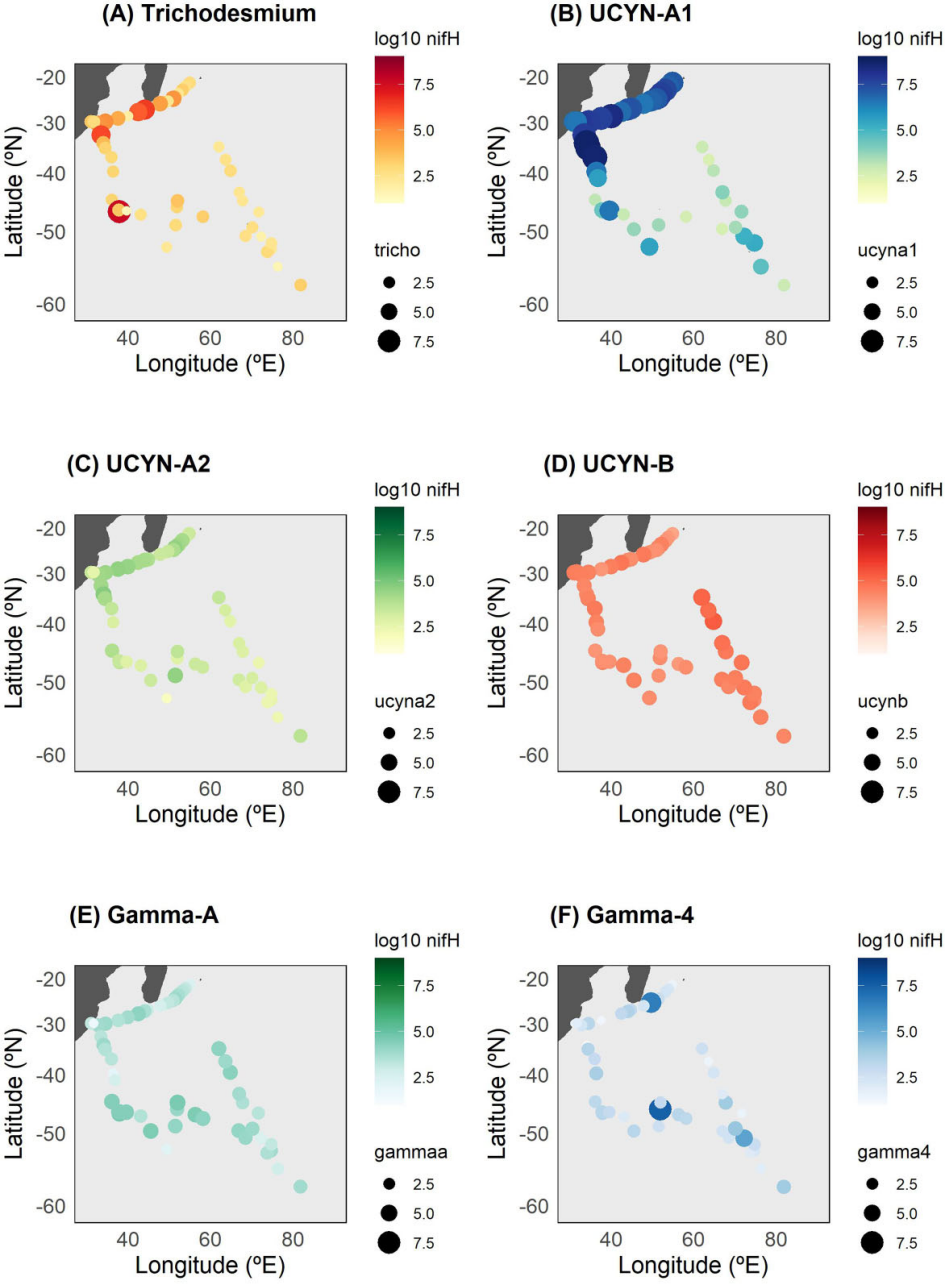
n*if*H gene abundance (log_10_  *nif*H gene copies l^−1^) of (A) *Trichodesmium*, (B) UCYN-A1, (C) UCYN-A2, (D) UCYN-B, (E) Gamma-A, and (F) Gamma-4 assessed by qPCR in surface samples (5 m) along the cruise transect.

The qPCR amplification of cyanobacterial and NCDs *nif*H phylotypes revealed substantial variability in both their distribution and abundance across the northern and southern regions of the fronts (Fig. 3). Cyanobacteria were more abundant north of the fronts, while NCDs dominated south of the fronts. Notably, our results showed a co-occurrence of UCYN-A1 and *Trichodesmium nif*H gene counts north of the fronts, particularly in the Agulhas region (Fig. 3A and B). In contrast, UCYN-A2, UCYN-B, and Gamma-A displayed a more uniform distribution across the entire sampled area, irrespective of the position of the fronts (Figs 3C–E). Furthermore, UCYN-A1 showed an increased abundance under conditions characterized by low concentrations of nitrate and phosphate (0.07–0.17 µmol l^−1^ and 0.02–0.09 µmol l^−1^, respectively). Conversely, Gamma-4 was more abundant at stations south of the fronts with elevated nitrate (21.50–23.45 µmol l^−1^) and phosphate (1.45–1.58 µmol l^−1^) concentrations (Fig. 3F).

### Diazotroph community composition

**Figure 4.**
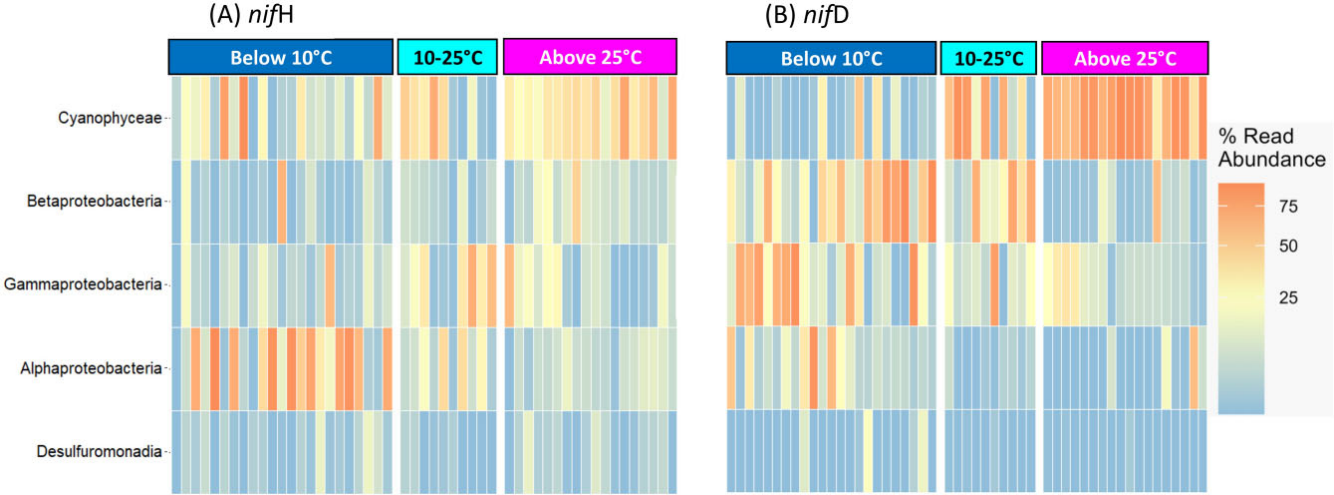
Heatmap representing most abundant ASVs across three temperature gradients for the (A) *nif*H gene, and (B) *nifD* gene. *nif*H groups have been reordered to facilitate comparison with *nif*D groups.

We assessed diazotrophic community composition using both *nifH* and *nifD* genes as biomarkers with 1 million reads per sample. This dual approach resulted in different spatial distributions of diazotroph groups, particularly when divided into NCDs and cyanobacterial diazotrophs. A total of 15,938,410 reads were retrieved, resulting in 1,915 ASVs from *nifH* amplicons, including representatives from 20 phyla, 38 classes, 68 orders, 115 families, and 146 genera. From the recovered *nifH* ASVs, 87% were NCDs and 13% were cyanobacterial diazotrophs. In waters with SST <10°C, the most abundant groups were betaproteobacteria from the families *Burkholderiaceae* and *Comamonadaceae*, followed by *Cyanophyceae* (*Trichodesmium*), thermodesulfobacteriota *(Desulfocarbo*), and gammaproteobacteria (*Vibrio*) (Fig. 4A). At SST levels between 10°C and 25°C Cyanophyceae were the prevailing diazotrophs, followed by gammaproteobacteria, thermodesulfobacteriota, and betaproteobacteria. In waters with SST >25°C, a community shift was observed from Pseudomonadota (proteobacterial) groups to Cyanobacteria, specifically unicellular Cyanobacteria (Candidatus *Atelocyanobacterium thalassa* and *Crocosphaera* sp.), along with *Trichodesmium* as the dominant diazotroph taxon (Fig. 4A). *nifD* gene amplicon sequencing resulted in 7,809,286 reads, resulting in 2,170 ASVs from 8 phyla, 12 classes, 26 orders, 47 families, and 46 genera, comprising 87% NCDs and 13% cyanobacterial diazotrophs (Fig. S4, Supplemental files). Based on *nifD* amplicons, betaproteobacteria dominated the community at SST <10°C, followed by gamma- and alphaproteobacteria (Fig. 4B). Between 10°C and 25°C the cyanobacterium *Trichodesmium* was detected, but the diazotroph community was primarily dominated by beta- and gammaproteobacteria (Fig. 4B). Above 25°C the community was dominated by cyanobacteria, including *Trichodesmium, Crocosphaera, Candidatus Atelocyanobacterium, Richelia, Zehria, and Hydrocoleum*. NCDs exhibited higher relative prevalence across the entire transect as assessed by *nifH* amplicon sequencing, while the *nifD* amplicon sequencing approach only should a higher relative prevalence south of the STF (Fig. S5, Supplemental files).

The *nif*H amplicons captured a wide range of Cluster III diazotrophs, including taxa such as Bacteroidia, Terrimicrobiia, Chlorobiia, Desulfobacterota, Desulfovibrionia, and Desulfuromonadia (Fig. S6, Supplemental files). Conversely, the diversity of Cluster III diazotrophs was comparatively lower among the top 100 *nif*D ASVs (Fig. S7, Supplemental files). The *nif*D amplicons captured a more diverse cyanobacteria and gammaproteobacteria community. Specifically, within the cyanobacteria ASVs associated with *Richelia* and *Crocosphaera* were identified by *nif*D gene sequencing (Fig. S7, Supplemental files), whereas these diazotrophs were not detected by *nif*H gene amplification (Fig. S6, Supplemental files). However, through *nif*H gene sequencig we found some unicellular Cyanobacteria that showed genetic similarity to UCYN-A1 and -A2 previously identified in the Pacific and Antarctic oceans (Fig. S6, Supplemental files).

The relative contribution of ASV counts by *nifH* and *nifD* gene sequencing at the three subregions clustered by SST (Supplemental files) showed that *Trichodesmium, Crocosphaera*, UCYN-A1, and *Richelia* were the prevailing diazotroph taxa north of the fronts (>25°C), with both *nifH* and *nifD* amplicon analysis approaches (Fig. S8, Supplemental files). Regarding NCDs, both analyses differed: the *nifH* amplicon data highlighted the prevalence of alphaproteobacteria at SST <10°C, beta, and zetaproteobacteria, along with desulfobacterota and thermodesulfobacteriota north of the fronts (>25°C), and gammaproteobacteria and clostridia in the 10–25°C temperature range. The *nifD* analysis revealed a predominance of alpha-, beta-, gammaproteobacteria, and thermodesulfobacteriota at SST < 10°C. In contrast, zetaproteobacteria were more abundant in the temperature range of 10–25°C.

The relative contribution of ASV counts by *nif*H and *nif*D amplicon sequencing showed distinct distribution patterns of major diazotroph groups (Supplementary data on figshare). *nif*H showed *Trichodesmium* was most prevalent (66%) in samples >25°C, while UCYN-A1 dominated at temperatures <25°C (57%) but decreased sharply in the 10°C–25°C range (41%) and in waters <10°C (2%). *Crocosphaera* dominated in waters >25°C (59%) but declined to 28% in waters with SST between 10°C and 25°C, and represented only 13% in waters <10°C. *Richelia* was primarily observed above 25°C (88%) but dropped to 12% in the 10°C–25°C range and was absent below 10°C. Alphaproteobacteria was distributed across all temperature gradients, with 44% below 10°C, 29% at 10°C–25°C, and 27% above 25°C. Betaproteobacteria were prominent at temperatures above 25°C (66%), while gammaproteobacteria varied from 26% above 25°C to 59% at 10°C–25°C and 15% below 10°C. Zetaproteobacteria were prevalent above 25°C (83%) but less so at 10°C–25°C (7%) and below 10°C (10%). Desulfobacterota dominated at temperatures above 25°C (97%), while Clostridia were highly prevalent (95%) at 10°C–25°C. Euryarchaeota exhibited a preference for the 10°C–25°C temperature range (68%).

*nif*D sequencing showed that *Trichodesmium* predominated with 75% abundance in waters above 25°C. Again based on *nif*D sequencing, UCYN-A1 represented 61% of the sequences in waters with SST>25°C, 39% in for waters in the 10°C–25°C range, and was absent in waters <10°C. *Crocosphaera* dominated with 83% abundance above 25°C. *Richelia* was mostly above 25°C (96%). Alphaproteobacteria distributed as 43% above 25°C, 2% in 10°C–25°C, and 55% below 10°C. Betaproteobacteria dominated in the below 10°C range with 62% abundance. Gammaproteobacteria exhibited varying distributions, with 67% in samples below 10°C. Zetaproteobacteria constituted 21% of ASV counts above 25°C, 66% at 10°C–25°C, and 13% below 10°C. Thermodesulfobacteriota highly dominated at below 10°C with 87% counts. These findings shed light on the temperature-dependent distribution patterns of major diazotroph groups.

### Environmental drivers of diazotroph community composition

Correlation analysis between *nifH* gene counts and environmental parameters revealed positive relationships of UCYN-A1 and -A2, as well as *Trichodesmium*, with salinity and SST (Fig. S9, Supplemental files). Additionally, inverse correlations were observed between the gene counts of these groups and nitrate, phosphate, nitrate to phosphate (N/P) ratios, and silicate concentrations, particularly for UCYN-A2 *nif*H gene counts (*r* = −0.72; Fig. S9, Supplemental files). The analysis revealed that dMn exhibited a significant positive correlation with the abundance of *Trichodesmium* (r0.74), UCYN-A1 (*r* = 0.64), the *nifD* cyanobacterial community (*r* = 0.94), as well as with N_2_ fixation rates (*r* = 0.75) (Figs S9 and 10, Supplemental files). Dissolved Pb showed significant strong correlations with abundance of *Trichodesmium* (*r* = 0.91), UCYN-A2 (*r* = 0.69), and *nifD* cyanobacterial community (*r* = 0.76). Moreover, dCo exhibited a robust correlation with the abundance of *Trichodesmium* (*r* = 0.80), and dNi negatively correlated with N_2_ fixation (*r* = −0.86) and *nif*D (*r* = −0.63) cyanobacterial community (Fig. S9, Supplemental files).

**Figure 5.**
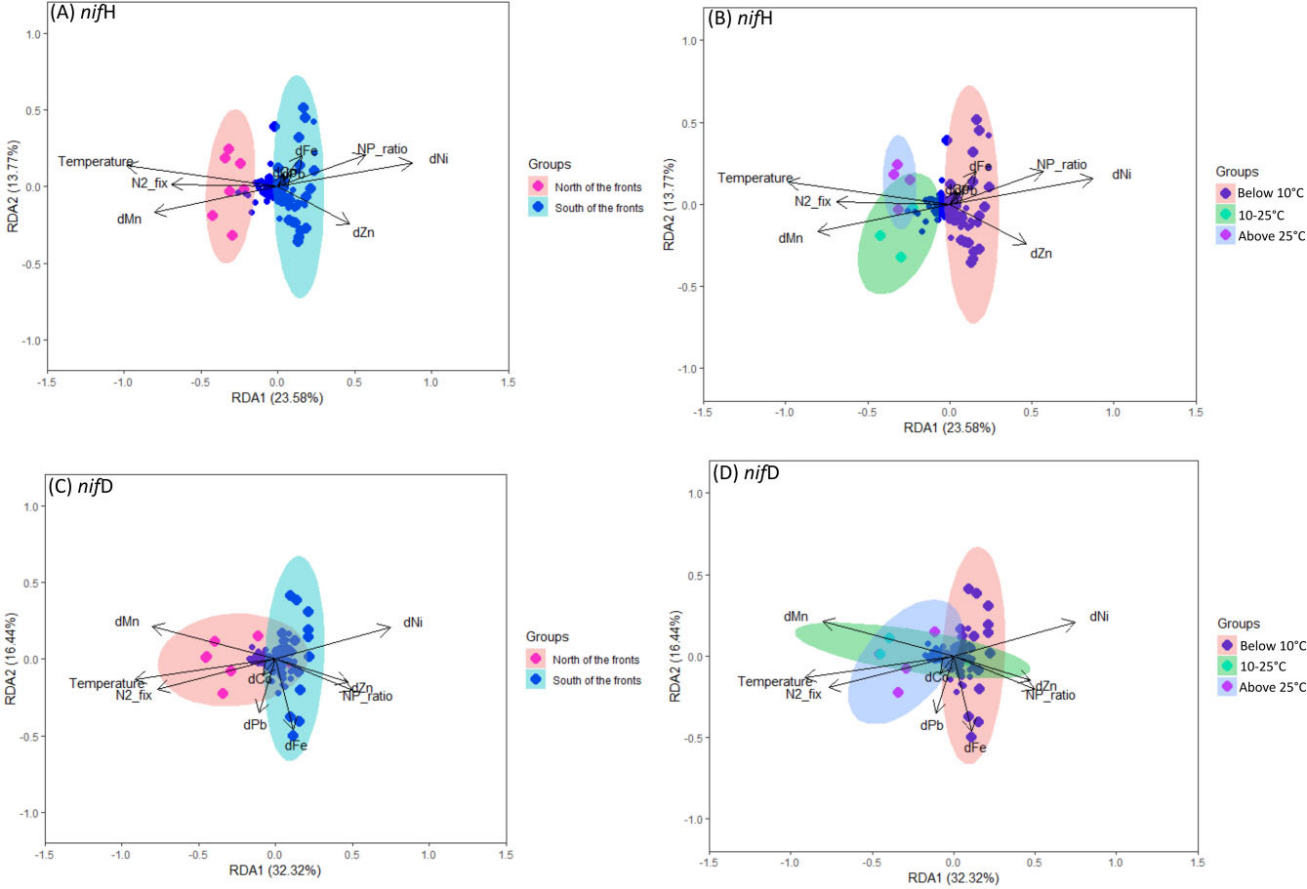
Redundancy analyses showing the influence of environmental variables and N_2_ fixation rates on diazotroph community composition based on (A and B) *nif*H genes and (C and D) *nif*D genes, grouped as north and south of the fronts or by temperature cluster, respectively.

The distinctive distribution patterns observed among ASVs and environmental factors across various temperature ranges and location with respect to the fronts underscored the temperature-dependent dynamics of the diazotrophic community (Fig. 5). Salinity and SST exhibited a positive correlation on *nifD* cyanobacterial community composition (Fig. S9, Supplemental files). Concurrently, the composition of the NCDs community displayed significant correlations with N/P ratios (*r* = 0.92) (Fig. S9, Supplemental files). At SST<10°C, particularly in the south of the fronts, the distribution of *nifH* ASVs was strongly associated with environmental parameters, including the N/P ratio, dNi, and dZn concentrations, driving community composition (Fig 5). Furthermore, *nifD* ASVs in the same temperature range displayed associations not only with the aforementioned variables but also with dFe concentrations (Fig. 5). Conversely, in regions characterized by higher temperature gradients, specifically to the north of the fronts (ranging from 10°C to 25°C and >25°C), the distribution of ASVs appeared to be associated with temperature and dMn concentration (Fig. 5). Additionally, the ASVs detected at these higher temperatures were key drivers of the observed N_2_ fixation activity (Fig. 5).

## Discussion

### SIO fronts divide diazotroph communities

Our results show a clear north–south divide, with substantial N_2_ fixation rates north of the fronts and mostly undetectable rates south of the fronts (Fig. 1C). While N_2_ fixation was generally not detected south of the fronts during our study, previous studies measured rates up to 1.97 nmol N l^−1^ d^−1^ during the same season in this region (Hörstmann et al. 2021). The SIO fronts are however not impermeable, with meandering and cross-front heat and tracer exchange due to baroclinic instabilities and bathymetry patterns (Chapman et al. 2020). Hence, we cannot rule out that previous significant N_2_ fixation activity measurements in previous studies may have been influenced by diazotroph transport across the fronts and/or transfer of trace metal rich waters into the reactive nitrogen poor waters of the IO subtropical gyre.

While cyanobacteria dominated north of the fronts, we highlight the detection of *Trichodesmium* south of the fronts (Fig. 4). The presence of *Trichodesmium* on both sides of the fronts may result from advection, as observed for other organisms with positive buoyancy (Fraser et al. 2022). Alternatively, the presence of *Trichodesmium* south of the fronts may be explained by their poleward migration, indicating potential adaptation to colder waters. While capable of growth at temperatures below 20°C (Rivero-Calle et al. 2016), *Trichodesmium* may experience compromised N_2_ fixing efficiency under such conditions. Numerical advection modelling indicates that *Trichodesmium* communities can endure for over 3.5 months at temperatures below previous expectations (Rees et al. 2016), suggesting their occupancy of a distinct niche relative to other *Trichodesmium* species as a cold- or low-light-adapted variant from the SIO.

South of the fronts, the diazotroph community was dominated by NCDs (Figs 3 and 4), mainly composed of alpha-, beta-, gammaproteobacteria, and thermodesulfobacteriota (Fig. 4). Alphaproteobacteria are adapted to thrive in cold, open-ocean environments (Verde et al. 2016), likely explaining their significant presence at SST levels below 10°C in our study region (Fig. 4, Fig. S8, Supplemental files). Below 10°C, alphaproteobacteria were mostly represented by the order Rhodobacterales and Hyphomicrobiales, which have a chemoheterotrophic metabolism and are capable of sulphur oxidation and carbon reduction along with N_2_ fixation (Pujalte et al. 2014). Thermodesulfobacteriota, known for their sulfate-reducing abilities, showed a dominant presence below 10°C (Fig. S8, Supplemental files). The Southern Ocean holds significant importance in the recirculation of climate-active trace gases, including dimethyl sulfoxide and methane (Thurber et al. 2020). This could be linked to the important presence of methanotrophs and sulfate reducers south of fronts. Likewise, beta- and gammaproteobacteria were mostly detected at temperatures below 10°C range (Fig. S8, Supplemental files).

Even if NCDs were dominant south of the fronts, we could not find evidence of active N_2_ fixation (Fig. 2). A similar predominance of NCDs together with undetectable rates have been reported from different regions, suggesting that NCDs may not contribute to N_2_ fixation significantly or may not be active at all (Turk-Kubo et al. 2014, Moisander et al. 2017). However, recent evidence showed N_2_ fixation active NCDs on suspended particles in the North Pacific subtropical gyre (Harding et al. 2022). N_2_ fixation by NCDs may respond to different conditions than those favouring cyanobacterial diazotrophs (Turk-Kubo et al. 2022), particularly the availability of labile dissolved organic matter in light of the lack of the photosynthetic machinery to generate ATP for N_2_ fixation (Benavides et al. 2015, Riemann et al. 2022). Moreover, N_2_ fixation in NCDs is likely intermittent and responds to other favouring conditions such as the presence of low oxygen microzones (e.g. in particles) and low reactive nitrogen concentrations (Bombar et al. 2016, Bianchi et al. 2018, Chakraborty et al. 2021). Hence, we cannot fully rule out that NCDs fix N_2_ in the SIO when conditions are favourable.

The observed patterns in the diazotroph community composition and N_2_ fixation rates within the studied region are closely intertwined with the prevailing ecological factors. The high salinity and elevated temperatures observed north of the fronts appeared to promote the proliferation of cyanobacterial diazotrophs, likely due to their adaptation to such conditions (Furbo Reeder et al. 2022). Conversely, the high inorganic nutrient concentrations and high N/P ratio values south of the fronts correlated negatively with the abundance of cyanobacterial diazotrophs and N_2_ fixation activity (Fig. S9, Supplemental files). This confirms previous findings of cyanobacterial diazotrophs being mostly favoured in oligotrophic environments (Zehr 2011). While previous studies have focused on dFe as the main trace metal impacting cyanobacterial diazotrophs, we found strong positive correlations between dMn and SST, cyanobacterial diazotroph abundance and N_2_ fixation rates (Fig. 5, Fig. S9, Supplemental files), suggesting a potential synergistic effect between these factors. Higher temperatures may enhance the metabolic activity of cyanobacterial diazotrophs, while dMn availability could serve as an essential micronutrient for their growth (Browning et al. 2021). In contrast, inorganic nutrients and the trace metals dNi and dZn were statistically related to the abundance of NCDs, suggesting that these diazotrophs may thrive in environments with different nutrient stoichiometry than cyanobacteria. NCDs can have various metabolic capabilities including sulfate reduction, nitrate reduction, thiosulfate oxidation, and hydrocarbon and organic matter degredation (Bentzon-Tilia et al. 2015, Turk-Kubo et al. 2022). Trace metals like dCo, dMn, dNi, and dZn are key elements that boost the sulfate redcution and hydrocarbon and organic matter degradation abilities of NCDs (Luek et al. 2017). Increasing dNi concentrations also have been shown to enhance cellular superoxide dismutase activities and N_2_ fixation rates (Ho 2013). These patterns suggest that further studies examining the trace metal regulation of N_2_ fixation, beyond dFe are needed.

### Hotspots of diazotroph activity in the SIO

Besides the main north–south frontal divide, we found two particular hotspots of diazotrophic activity: the waters around the southern tip of Madagascar and the Agulhas Current. Previous studies have shown significant N_2_ fixation in these regions of southwest IO (Shiozaki et al. 2014, Fernández-Castro et al. 2015, Hörstmann et al. 2021, Metzl et al. 2022, Sato et al. 2022). Metzl et al. (2022) reported N_2_ fixation rates up to 18.26 nmol N l^−1^ d^−1^ coinciding with an interannually variable feature known as the “Madagascar Bloom” (Longhurst 2001). Similarly, modelling approaches have reported high N_2_ fixation activity in correlation with Madagascar Bloom events (Tang et al. 2019). This phenomenon is associated with conditions favourable for N_2_ fixation including increased water column stratification, dFe availability during the rainy season due to island runoff, as well as mesoscale circulation (Metzl et al. 2022, Raes et al. 2022). However, while the strength of the Madagascar Bloom was much lower during our study than in previous ones where high N_2_ fixation rates were reported (Fig. S11, Supplemental files), we measured rates up to 13.96 nmol N l^−1^ d^−1^ around Madagascar (Fig. 2A). The high dMn concentrations and optimal SST conditions (21.79°C–29.03°C) around Madagascar during our cruise could have potentially driven these high N_2_ fixation rates. *Trichodesmium* was one of the most abundant diazotrophs south of Madagascar during our study, with up to 2.5 × 10^6^  *nif*H gene copies l^−1^ (Fig. 3), as expected from previous studies showing high abundance of this cyanobacterium in the region (Poulton et al. 2009, Srokosz and Quartly 2013), despite low dFe availability (Wilson and Qiu 2008). However, we also found a concomitant high abundance of UCYN-A1 (up to 5 × 10^8^  *nif*H gene copies l^−1^; Fig. 3), suggesting these diazotrophs were also contributing to the high rates measured.

The Agulhas current is characterized by dynamic processes, including eddies and upwelling events, which can lead to the entrainment and transport of nutrient-rich waters from deeper layers to the surface (Lutjeharms et al. 2000). Nutrient inputs combined with favourable physical conditions such as increased sunlight availability and warmer temperatures may create a favourable environment for the growth and proliferation of diazotrophic cyanobacteria like *Trichodesmium*. Isotopic nitrate signatures in the greater Agulhas area suggest active N_2_ fixation takes place here (Marshall et al. 2023), but not in its northern and eastern branches (Sigman and Fripiat 2019). N_2_ fixation in this region may be sustained by high dFe concentrations, primarily resulting from the resuspension of shelf sediments and atmospheric deposition (Grand et al. 2015). Moreover, the western Mozambique Channel shelf contributes to a phosphorus excess (>0.3 μM), potentially enhancing N_2_ fixation in the Agulhas current (Marshall et al. 2023).

### Biogeochemical implications

Significant alterations in the global N_2_ fixation budget (from 74 ± 7 to 223 ± 30 Tg N yr^−1^) have primarily been ascribed to diazotrophy in the IO (Luo et al. 2012, Shao et al. 2023). The total nitrogen input through N_2_ fixation in the IO subtropical gyre has been estimated to range from 1.26 to 2.19 Tg N yr^−1^, and from 2.17 to 2.27 Tg N yr^−1^ in the SIO (Supplemental files). Together, the IO subtropical gyre and the SIO contribute more fixed N_2_ to the global IO than its other sub-basins, i.e. the Arabian Sea, Bay of Bengal, Equatorial IO, and Eastern IO (Chowdhury et al. 2023). Still, the availability of diazotrophy data in the IO is much lower than in the North Atlantic and Pacific oceans. This shortfall in N_2_ fixation data and the high variability of the data available to date may have significant biogeochemical implications for global nitrogen budget calculations. The dynamic nature of N_2_ fixation, as evidenced by variations in estimates, suggests a multifaceted interplay of factors. Regional disparities in nutrient availability, the distribution of diazotrophs, and the influence of ocean dynamics features such as currents and upwelling events, are potential drivers behind these differences. Furthermore, the variability in N_2_ fixation can propagate through the marine food web, impacting primary productivity and fish landings. Therefore, gaining a comprehensive understanding of N_2_ fixation in the IO, especially in the less-studied regions of IO subtropical gyre and the SIO, is crucial for refining our knowledge of global nitrogen cycling and its far-reaching ecological consequences.

## Conclusions

The SIO fronts imprinted a clear latitudinal divide in nutrients and trace metal availability, coinciding with a sharp differentiation of the diazotroph community composition and associated N_2_ fixation activity in the region. North of the fronts, cyanobacterial diazotrophs dominated, likely driving the high N_2_ fixation activity observed. Conversely, south of the fronts NCDs dominated while no significant N_2_ fixation activity was observed. Our findings suggest that the presence of certain trace metals, and particularly dMn, may influence the activity and composition of the diazotroph community, while others such as dFe, usually regarded as key for diazotrophy, did not seem to have an impact in the SIO diazotroph community as suggested by statistical analyses. Additionally, our results highlight the potential of the *nif*D gene as a better descriptor of NCDs taxonomy compared to the more commonly used *nif*H gene. Projections of net primary production increasingly diverge in Earth system models (Tagliabue et al. 2021), in part due to the parameterization of N_2_ fixation under climate change, with the tropical IO being among the most uncertain regions (Bopp et al. 2022). Hence, focusing future N_2_ fixation research in the IO is needed to constrain future net primary productivity projections. Because N_2_ fixation activity differs greatly between diazotroph groups, community composition data is needed along with N_2_ fixation rate measurements. Our data provides a comprehensive insight into N_2_ fixation activity and diazotroph community composition in the SIO, contributing to filling this gap.

## Supplementary Material

fiae095_Supplemental_Files
