# Supplementary material for: Fronts divide diazotroph communities in the Southern Indian Ocean
Source: FEMS Microbiol Ecol. 2024 Jul 11;100(8):fiae095. doi: 10.1093/femsec/fiae095 (PMC11245648; doi:10.1093/femsec/fiae095)
Supplement: fiae095_Supplemental_Files [file fiae095_supplemental_files.zip › Chowdhury_et_al_supplemental_file_03072024.docx]

**Supplemental Files**

This file contains:

Supplemental Methods

Supplemental Results

Supplemental Discussion

Supplemental Figures S1-S13

Supplemental References

**Supplementary Methods**

*N_2_ fixation and primary production rates*

N_2_ fixation rates were calculated according to (Montoya *et al.* 1996) using the following equation:

$$NFR = \frac{{A_{PO^{15}N, final} - A_{PO^{15}N, control}}}{A_{{15N}_{2}, incubation}- A_{{15N}_{2}, natural}}\times\frac{PON}{t}$$

With NFR, the N_2_ fixation rate, $A_{PO^{15}N, final}$, the ^15^N isotopic abundance measured in the particulate matter at the end of the incubation, $A_{PO^{15}N, control}$, the ^15^N isotopic abundance measured in the particulate matter in the control samples, $A_{{15N}_{2}, incubation}$, the ^15^N isotopic abundance measured in the dissolved N_2_ pool during the incubation, $A_{{15N}_{2}, natural}$, the ^15^N isotopic abundance in the dissolved N_2_ pool of the natural background (i.e. 0.3666 atom%), $PON$, the particulate organic nitrogen concentration, and t, the incubation time. Since no patterns were detected in $A_{PO^{15}N, control}$ over the course of the cruise, the average of all measured values was used for rate calculations at all stations (Fig. S1). For each station, N_2_ fixation was considered as detected when:

$A_{PO^{15}N, final}\geq$ $A_{PO^{15}N, control}+3 \times A_{PO^{15}N, control}$

Out of 51 measurements, 23 are above the limit of detection. Minimum quantifiable rates (MQR) were also calculated based on error propagation of the replicates as proposed by (White *et al.* 2020). N_2_ fixation rates and MQR are available in Figshare (<https://doi.org/10.6084/m9.figshare.24799011.v2>).

*DNA sampling, extraction, and nif*H*/nif*D *gene sequencing*

All PCRs were performed in 25 µl reactions with 5 µl MyTaq buffer (Bioline, London, United Kingdom), 1 µl of 10 µM forward and reverse primers, 4 µl of template DNA, 1.25 µl of 25 mM MgCl_2_, 0.5 µl of 10 mg/ml BSA, 0.5 unit of MyTaq DNA polymerase, and 12 µl of PCR grade water. The *nif*H PCR conditions were initial denaturation at 94°C for 2 min, followed by 31 cycles of final denaturation at 94°C for 1 min, annealing at 54°C for 1 min, and elongation at 72°C for 1min 30 seconds, with final elongation at 72°C for 7 min. To amplify *nif*D genes the first round of PCR was conducted by using the primers nifD820F (5'-CAC TGC TAY CGB TCG ATG AAC TAC-3’) and *nif*D 1389R (5ˈ-GAT GTC RCG SGC GAA GAT-3ˈ) followed by the second round of PCR by using *nif*D820F and *nif*D 1331R (5ˈ-CAG GAG TGC ATY TGV CGG 3ˈ) (McRose *et al.* 2017). PCR conditions for *nifD* amplification were initial denaturation at 94°C for 2 min, followed by 31 cycles of final denaturation at 94°C for 1 min, annealing at 55°C for 1 min, and elongation at 72°C for 30 seconds, and with final elongation at 72°C for 5 min.

*Diazotroph abundance quantification*

The abundance of *nif*H genes was quantified using TaqMan-specific quantitative PCR (qPCR) assays with previously published primers (Steward *et al.* 2004; Church *et al.* 2005; Moisander *et al.* 2008; Halm *et al.* 2012; Thompson *et al.* 2014). Four groups of cyanobacteria (*Trichodesmium*, UCYN-A1, UCYN-A2, and UCYN-B) and two groups of NCDs (Gamma-A and Gamma-4) were targeted. Standards for qPCR assays were produced from linearized plasmids containing the dilute with sequences of the respective group of diazotrophs were ordered from Genewiz (Leipzig, Germany), and Plasmid linearization was performed with the restriction enzyme HindIII to prepare standard curves for each of target diazotroph groups. After linearization, plasmids were quantified by Picogreen and diluted to prepare standards from 10^9^ to 10^1^ gene copies. Extracted DNA was normalized to 2 ng µl^-1^ and 1 µl of the template was used for qPCR analysis. The qPCR reactions had a total volume of 12.5 µl of qPCR, consisting of 1 µl (2 ng µl^-1^) of template DNA, 6.125 µl TaqMan qPCR buffer (Applied Biosystems, Villebon Sur Yvette, France), 0.5 µl of 10 µM forward and reverse primers (HPLC purified, Eurofins, Nantes, France), 0.25 µl probe at 10 µM, 4 µl PCR grade water and 0.125 µl of 10 mg ml^-1^ bovine serum albumin. qPCR program was 2 min at 50ºC, 10 min at 95ºC continued by 45 cycles of 30 s at 95ºC and 1 min at 60ºC (for UCYN-A2, 1 min at 64ºC). We used standard dilutions from 10^7^ to 10^1^ gene copies and samples and no-template controls (NTCs) in duplicate. NTCs did not show any amplification and the efficiency was 99 to 125%. The qPCR assays were run on a CFX96 Real-Time System thermal cycler (BioRad, Marnes-la-Coquette, France). Inhibition tests were carried out on all samples and each primer-probe set by adding the 2 µl of the 10^5^ copy standard to each sample. No inhibition was observed. The limit of detection (LOD) and detected but not quantifiable (DNQ) for the qPCR was 1 and 8 gene copies per reaction volume, respectively.

*Comparing nifH and nifD diazotroph database annotation*

*nifH* sequences with matching taxonomy to the *nif*D database were extracted from the *nif*H database v2.0.5 (Moynihan and Reeder 2023). Filtered sequences matching taxonomy level at phylum, class, and order were used to cross-compare and examine the diazotroph community composition using both *nif*H and *nif*D amplicons sequencing. We assessed the accuracy of taxonomic annotation by the bootstrap method from kingdom to genus level.

We assessed the robustness of taxonomic annotations by evaluating the strength of annotations for the top 100 ASVs using five distinct databases: *nif*D database, *nif*H database, *nif*H similar phylum, class, and order level databases. By comparing the taxonomic assignments across various samples, we determined the reliability and precision of taxonomic annotation for each ASV retrieved by *nif*H and *nif*D amplicon sequencing. Relative contributions of cyanobacteria and NCDs involved assessing the taxonomic classification of ASVs affiliated with cyanobacteria and NCDs and quantifying their respective contributions to the community composition. These approaches provide a comprehensive understanding of the taxonomic composition and functional contributions of diazotrophs within the studied ecosystem by dual amplicon approaches.

**Supplemental Results**

*Diazotroph abundance*

The distribution of most diazotrophs was impacted by the position of the fronts (Fig. 3). The abundance of *Trichodesmium* ranged from 8.3x10^1^ to 2.5x10^6^ *nif*H gene copies l^-1^ of seawater, north of the front, and from 0.36 x 10^1^ to 4.2 x 10^7^ *nif*H gene copies l^-1^ of seawater, south of the front, with isolate peak abundances at station 860, and 868, south of Madagascar and station 875, near the Marion island (Fig. 3A). UCYN-A1 showed a similar distribution to *Trichodesmium*, ranging from 3.9 x 10^5^ to 5 x 10^8^ *nif*H gene copies l^-1^ of seawater, north of the front and 3.1 x 10^2^ to 3.2 x 10^6^ gene copies l^-1^ of seawater, south of the front (Fig. 3B). UCYN-A2, UCYN-B, and Gamma-A were instead more homogeneously distributed across the sampling area independently of the front (Figs. 3C-E), with abundances ranging between 4 x 10^2^ to 5 x 10^4^ *nif*H gene copies l^-1^ of seawater, and 3.2 x10^3^ to 5 x 10^4^ *nif*H gene copies l^-1^ of seawater, 4 x 10^1^ to 1.6 x 10^4^ *nif*H gene copies l^-1^ of seawater, and 6.3 x 10^1^ to 3.2 x 10^4^ *nif*H gene copies l^-1^ of seawater, 6.3 x 10^3^ to 2 x 10^5^ *nif*H gene copies l^-1^ of seawater, and 1.6 x 10^2^ to 4.7 x 10^4^ *nif*H gene copies l^-1^ of seawater, for UCYN-A2, UCYN-B, and Gamma-A, respectively. Finally, Gamma-4 was relatively homogeneously distributed, with some peak abundances of 3.2 x 10^7^ *nif*H gene copies l^-1^ of seawater, at stations 882, 857 (3.9 x 10^6^ *nif*H gene copies l^-1^ of seawater), and 893 (2.5 x 10^5^ *nif*H gene copies l^-1^ of seawater) (Fig. 3F).

*Trichodesmium* was detected at 49 of 51 stations (except for 873 and 884), with abundance ranging from 10^1^ to 10^7^ *nif*H gene copies l^-1^ of seawater. The highest abundance of *Trichodesmium* was detected at stations 860, 868, and 875, with more than 10^6^ gene copies l^-1^ of seawater (Fig. 3A). We found that UCYN-A1 was the most abundant diazotroph detected from this transect with marked variation in the north and south of the fronts (Fig. 3B). UCYN-A1 was detected from 45 stations (except 882-884 and 887-889), with the highest average gene copy number ranging from 10^2^ to 10^8^ gene copies l^-1^ of seawater. UCYN-A1 was most abundant at stations 862 and 869-871, where nitrate and phosphate levels were very low (0.009 and 0.04 - 0.08 µM, respectively). We observed that UCYN-A2 is uniformly distributed all over the transect (not detected from station 873) and ranged from 10^2^ to 10^4^ *nif*H gene copies l^-1^ (Fig. 3C). UCYN-B and Gamma A were most consistently detected from all the stations of the transect. The abundance of UCYN-B ranged from 10^2^ to 10^5^ *nif*H gene copies l^-1^ of seawater (Fig. 3D).

Among the NCDs, Gamma A represents a more uniform distribution, whereas Gamma 4 has a unique and most variable distributional pattern (Figs. 3E-F). Gamma 4 was detected from 46 stations out of 51 (except 853, 862, 868, 873, and 880), and a very wide range of distribution from 10^1^ to 10^6^ *nif*H gene copies l^-1^ of seawater. A higher abundance of Gamma A and Gamma 4 was detected from the colder waters (below 10^o^C, south of the fronts). UCYN-A1 is more prevalent when nitrate and phosphate concentrations are low, while Gamma 4 is more prevalent when nitrate and phosphate concentrations are high.

*Diazotroph community composition*

Analysis of the *nif*H gene across 51 DNA samples resulted in a total of 15,938,410 reads, from which 2,228 ASVs were retrieved. hmmsearch in HMMER yielded 1917 unique *nif*H sequences without rejections. Framebot identified 313 ASVs unable to overcome potential frameshifts. The attempt to detect *nif*H homologs (bchX, chlL, bchL, and parA) did not yield any positive identifications. A total of 1915 ASVs were classified into *nif*H phylogenetic clusters (Chien and Zinder 1996), following the categorization outlined by Frank et al. (2016), and were subsequently employed for further downstream analysis. The recovered *nif*H ASVs comprised 47% other NCDs excluding pseudomonadota (Proteobacteria), 40% Pseudomonadota, and 13% Cyanobacteria within the *nif*H sequences (Fig. S4). Within Cyanobacteria, approximately 62% were classified as unicellular Cyanobacteria (UCYN-A and -B), while 25% were identified as *Trichodesmium*. The remaining 13% represented other cyanobacterial taxa like *Richelia, Katagnymene, Leptolyngbya, Nostoc,* and *Hyella*. Among Pseudomonadota, Gammaproteobacteria are predominant (58%), followed by Beta (21%), Alpha (19%), Zetaproteobacteria (1%), and 2% as unclassified. The other NCD groups, mainly comprised of Thermodesulfobacteriota (54%), Bacillota/Firmicutes (11%), Verrucomicrobia (10%), Bacteroidota (4%), Lentisphaerota (4%), Desulfobacterota (3%), Planctomycetota (2%), and other taxa (Fig. S4). Thermodesulfobacteriota fraction comprised prominent sulfate-reducing diazotrophs, including *Desulfocarbo, Desulfopila, Desulfuromonas, Desulfovibrio,* and *Malonomonas*. Detected predominant gammaproteobacterial groups are *Amphritea, Agarivorans, Terdinibacter*, and *Vibrio*.

Amplicon sequencing of the *nif*D generated 7,809,286 reads from 51 samples. Within cyanobacteria, 64% were *Trichodesmium*, 20% were unicellular Cyanobacteria (UCYN-A and -B), and the remaining 16% represented other cyanobacterial taxa. The abundance of other NCDs by *nif*D (excluding pseudomonadota), was approximately six times lower than that observed in the *nif*H amplicon sequencing. Other NCDs are mainly represented by Thermodesulfobacteriota (7%). Pseudomonadota fraction was dominated by gamma (47%), followed by beta (29%), alpha (22%), and zetaproteobacteria (2%). Gammaproteobacterial groups *Stutzerimonas*, and *Acidihalobacter* were notable, while the Alphaproteobacterial order Rhodobacteraceae and Hyphomicrobiales were predominantly detected by *nif*D.

*Comparison nifH vs. nifD*

A phylum-level representation showed a similar trend for both *nif*H and *nif*D. Cyanobacterial diazotrophs at the phylum level represented 12% and 10% of the community in the *nif*H and *nif*D amplicons, respectively. At the phylum level, the entire NCD community was represented by 87% of the *nif*H sequencing and 90% of the *nif*D sequencing. Annotated ASVs with class-level comparisons showed a higher percentage of the *nif*D approach than the *nif*H approach for the Alpha- and Betaproteobacteria. At first glance, the diazotroph community appeared similar when analyzed using both the *nif*H and *nif*D methods. However, upon closer examination, pronounced variations become evident between the *nif*H and *nif*D amplicon sequencing approaches. Specifically, at the phylum level, the pseudomonadota is better represented by the *nif*D method than *nif*H. Conversely, the *nif*H approach effectively captured the community composition of other diazotroph groups such as Clostridia, which are known to have pseudo-*nif*H sequences (Mise et al., 2021). Digging further into the taxonomic classes within pseudomonadota, namely alpha, beta, gamma, and zetaproteobacteria, *nif*D demonstrates a superior representation compared to *nif*H.

Comparative non-metric multidimensional scaling (NMDS) analyses of *nif*H-based vs. *nif*D-based approaches showed a more pronounced separation of diazotroph communities by the *nif*D (Fig. S12). Shannon and Chao 1 indices showed higher temperatures (above 25°C) represented the highest diversity for both *nif*H and *nif*D (Fig. S13). Whereas the Simpson index showed diversity increased with temperature for *nif*H, diversity remained relatively stable for *nif*D (Fig. S7). The strength analysis of the *nif*D database revealed notable trends, indicating higher strength scores in more fine-scale taxonomic categories than all *nif*H databases (Fig. S14). As we moved towards finer taxonomic resolutions, the strength values remained consistently high for the *nif*D.

Regarding total read counts, the *nif*H exhibited more UCYN-A1, Alpha and Gammaproteobacteria, Clostridia, and Thermodesulfobacteriota. Conversely, the *nif*D displayed a greater abundance of *Trichodesmium, Crocosphaera, Richelia*, beta, and Zetaproteobacteria.

**Supplemental Discussion**

*A dual approach to study diazotroph community composition*

The observed distribution patterns of ASVs based on *nif*H and *nif*D genes by NMDS, provide valuable insights into the biogeography of diazotrophic communities. The divergent behaviors of *nif*H and *nif*D genes in response to temperature gradients hold significant implications for the study of diazotroph biogeography. The overlapping clusters of ASVs for the *nif*H gene across all three temperature ranges indicate a certain level of shared community composition. This result might suggest a certain degree of flexibility or adaptability among *nif*H-carrying organisms to thrive across a wide range of temperature conditions. On the other hand, the well-clustered ASVs for the *nif*D imply a stronger temperature-dependent effect on the distribution pattern. The distinct clustering suggests that *nif*D provides more specific responses to temperature, leading to more pronounced community shifts with changing temperatures. This increased sensitivity to temperature underscores the potential of the *nif*D gene as a robust indicator for studying diazotroph biogeography.

Given its clear responsiveness to temperature variations, the *nif*D gene holds promise as a valuable marker for tracking diazotrophic communities across diverse marine environments. A distinct latitudinal transition in the diazotroph community, as reflected in the relative contributions of cyanobacteria and NCDs to both community composition and abundance, is more effectively elucidated using *nif*D. Future diazotroph biogeography studies may prioritize the *nif*D gene as a key marker to uncover temperature-mediated distribution patterns and their ecological implications. Degenerated primers in *nif*H qPCR can lead to substantial mis-estimations (Gaby and Buckley 2017). Supplementing with *nif*D gene studies will aid in developing species-specific qPCR probes for more accurate quantification.

Strength analysis of taxonomic annotations for the *nif*H and *nif*D genes (Fig. S 14) shed light on the reliability and accuracy of taxonomic assignments across different taxonomic levels. The *nif*D database displays stronger performance at finer taxonomic resolutions, indicating increased precision and robustness in taxonomic assignments. Consistently high strength scores across phylum to genus levels affirm the suitability of the *nif*D amplicons for detailed diazotroph taxonomic investigations. The *nif*H database displayed relatively lower strength scores compared to the *nif*D database, suggesting potentially reduced accuracy in taxonomic annotations. However, when using the *nif*H similar phylum level, class level, and order level databases, the strength scores improved, indicating enhanced taxonomic resolution and reliability at these specific taxonomic levels.

These findings emphasize the importance of *nif*D as an appropriate marker for diazotroph biogeography and community composition. However, it is crucial to consider potential limitations and biases associated with the databases used, as well as the inherent variability and complexity of microbial communities. Future studies could explore the *nif*D amplicon as a potential marker gene for diazotroph community composition and identification and evaluate the consistency of taxonomic annotations across diverse microbial datasets to further enhance the reliability and comprehensiveness of taxonomic assignments in diazotroph communities. These findings underscore the suitability of the *nif*D as a marker for the biogeography of diazotrophs, enabling a more comprehensive understanding of the ecological roles and interactions.

**Supplemental Figures**


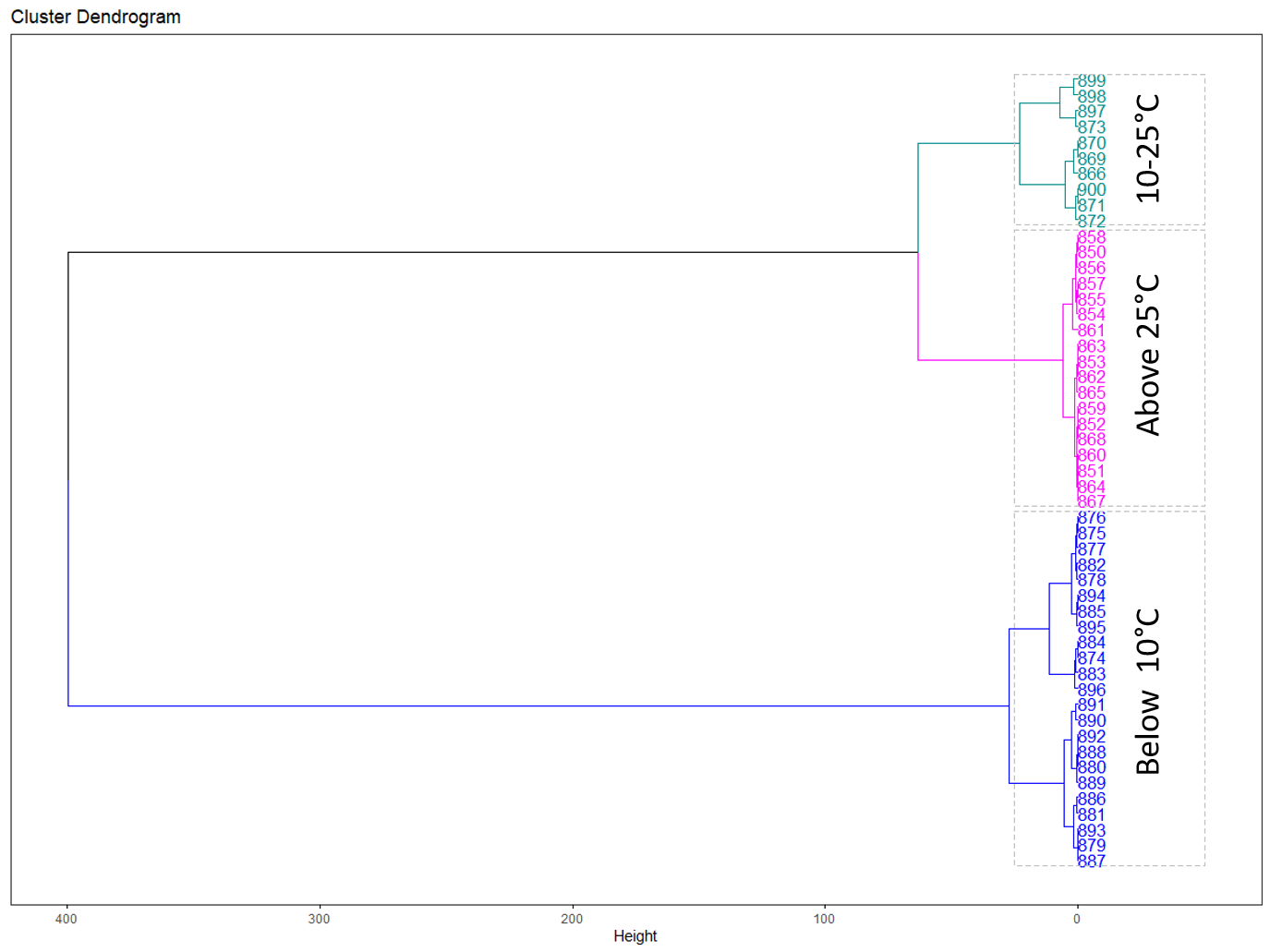


Fig. S1: Dendrogram clustering sampling stations by temperature: below 10°C including 23 stations (blue), 10-25 °C including 10 stations (cyan), Above 25°C including 18 stations (magenta).


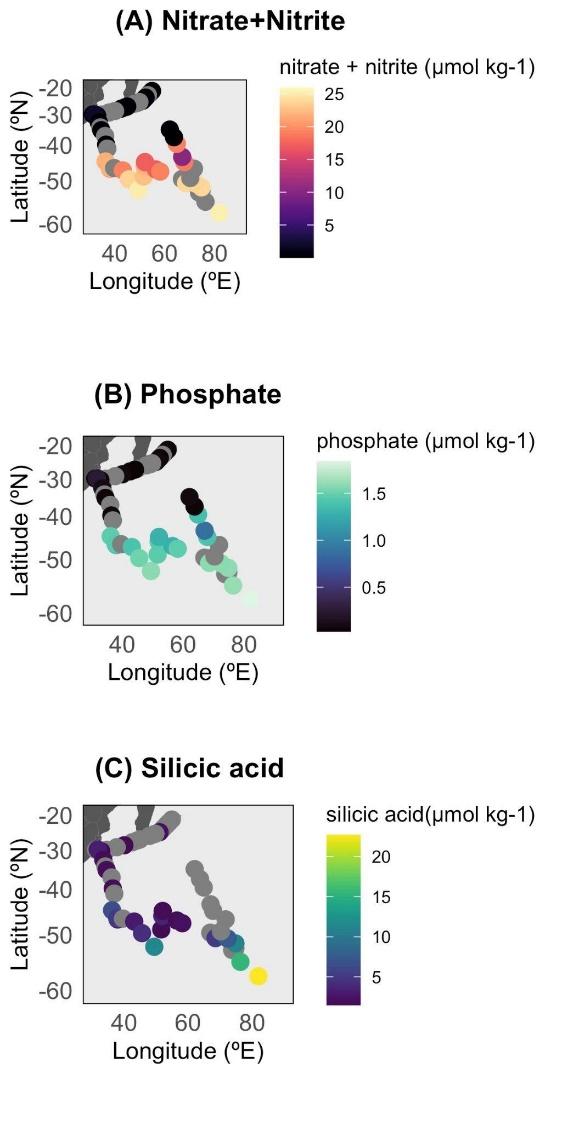


Fig. S2: Dissolved inorganic nutrient concentrations of the sampling stations along the SWINGS cruise transect.


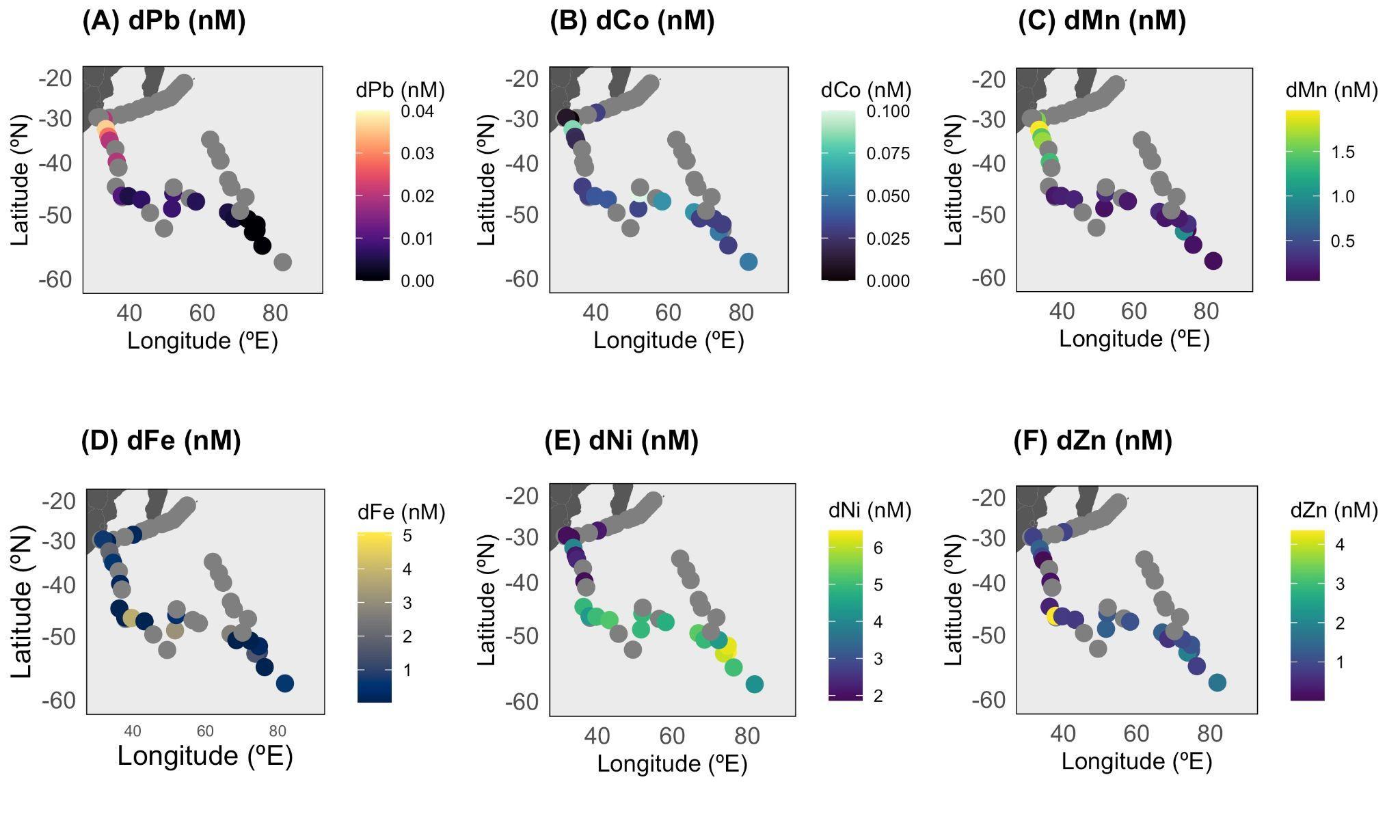


Fig. S3: Dissolved trace metal concentrations of the sampling stations along the SWINGS cruise transect.


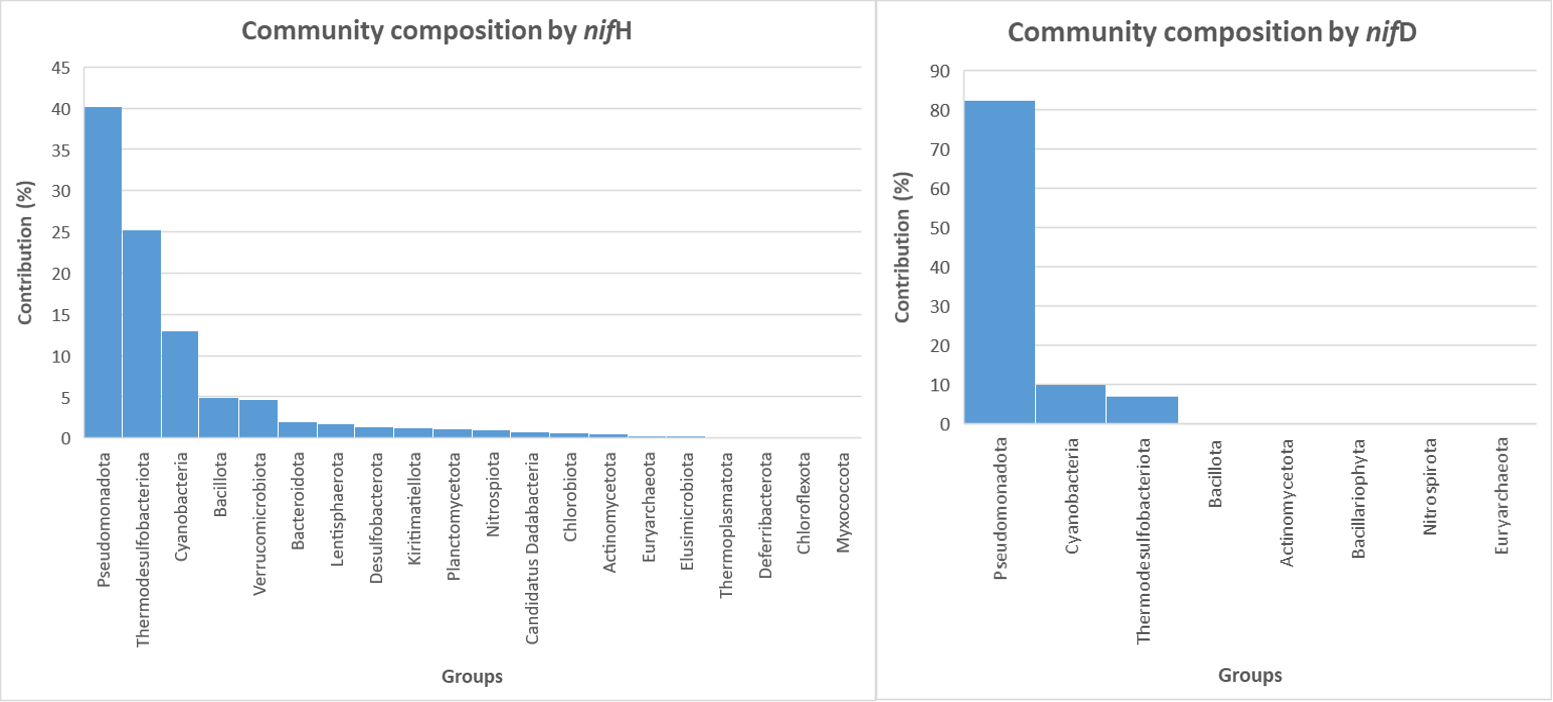


Fig S4: Contribution of various groups to diazotroph community composition based on *nif*H (left) and *nif*D (right) gene amplicon sequencing.


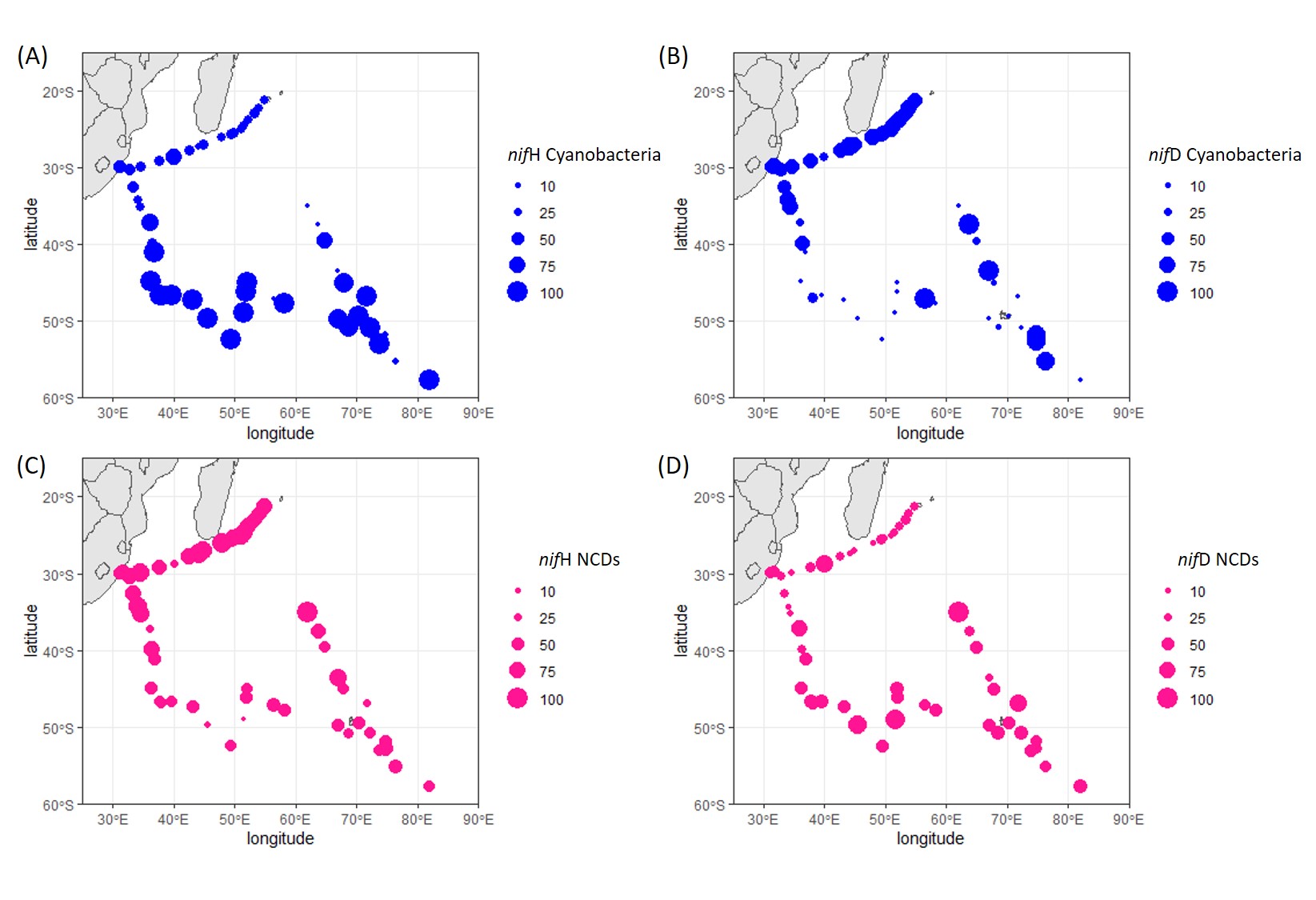


Fig. S5: Relative contribution (%) of Cyanobacteria based on (A) *nif*H and (B) *nif*D genes, and NCDs based on (C) *nif*H and (D) *nif*D genes to diazotroph community composition.

Added as a supplemental file for improved resolution

Fig. S6: *nif*H gene phylogenetic tree showing the relative abundance of the top 100 ASVs according to temperature clusters and sampling stations along the cruise transect.

Added as a supplemental file for improved resolution

Fig. S7: *nif*D phylogenetic tree showing the relative abundance of the top 100 ASVs according to temperature clusters and sampling stations along the cruise transect.


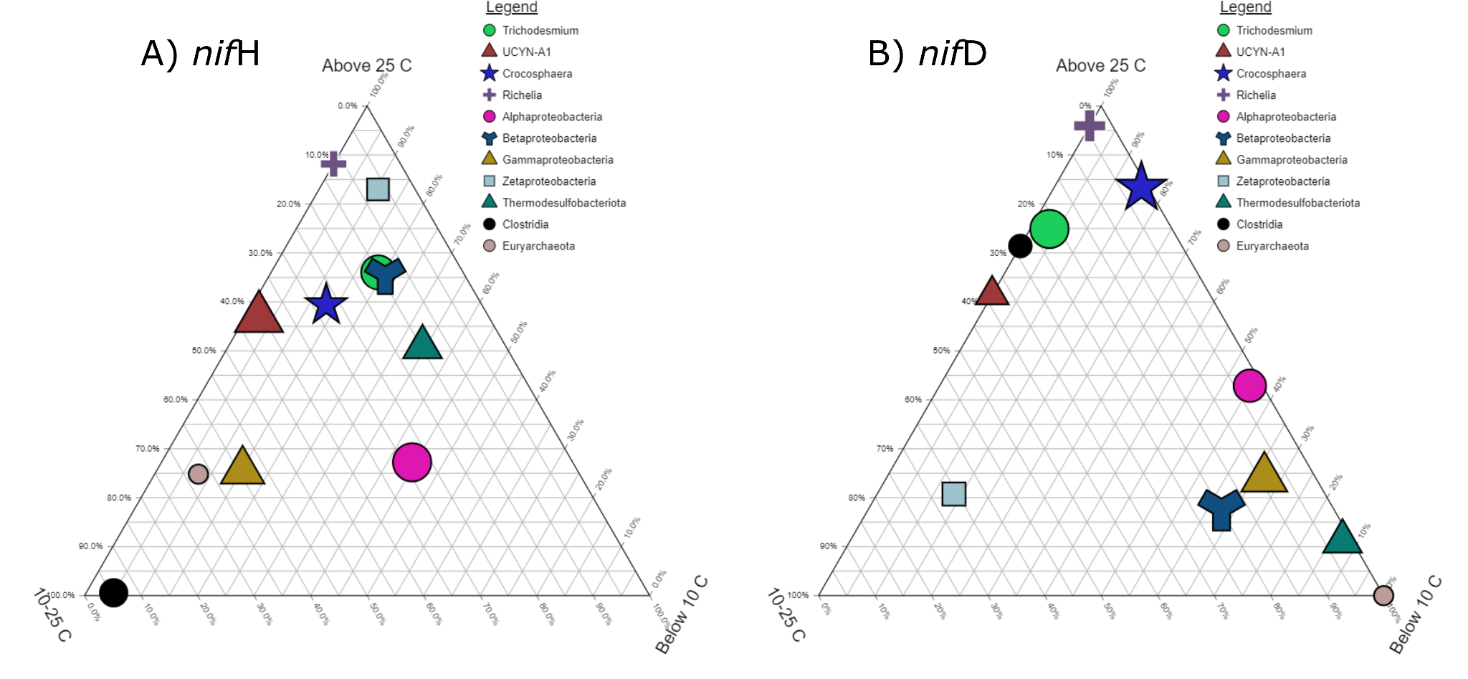


Fig S8: Exploring the diazotroph community's temperature preferences: ternary plots of (A) *nif*H and (B) *nif*D in three temperature clusters: below 10°C, 10-25°C, and Above 25°C.

**
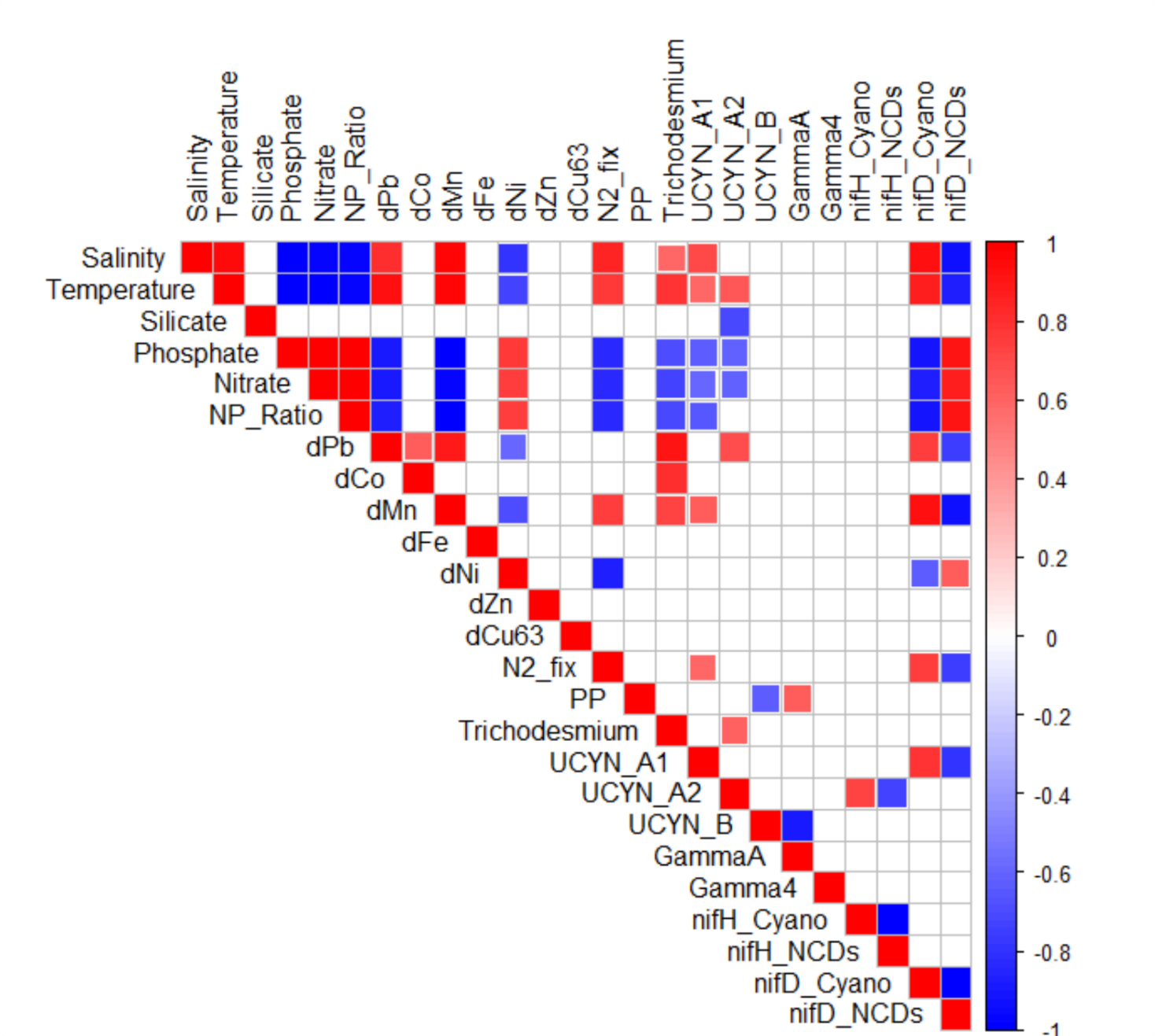
**

Fig. S9: Spearman correlations between environmental variables, diazotroph abundance based on *nif*H qPCR assays, and diazotroph community composition by *nif*H and *nif*D amplicon sequencing.


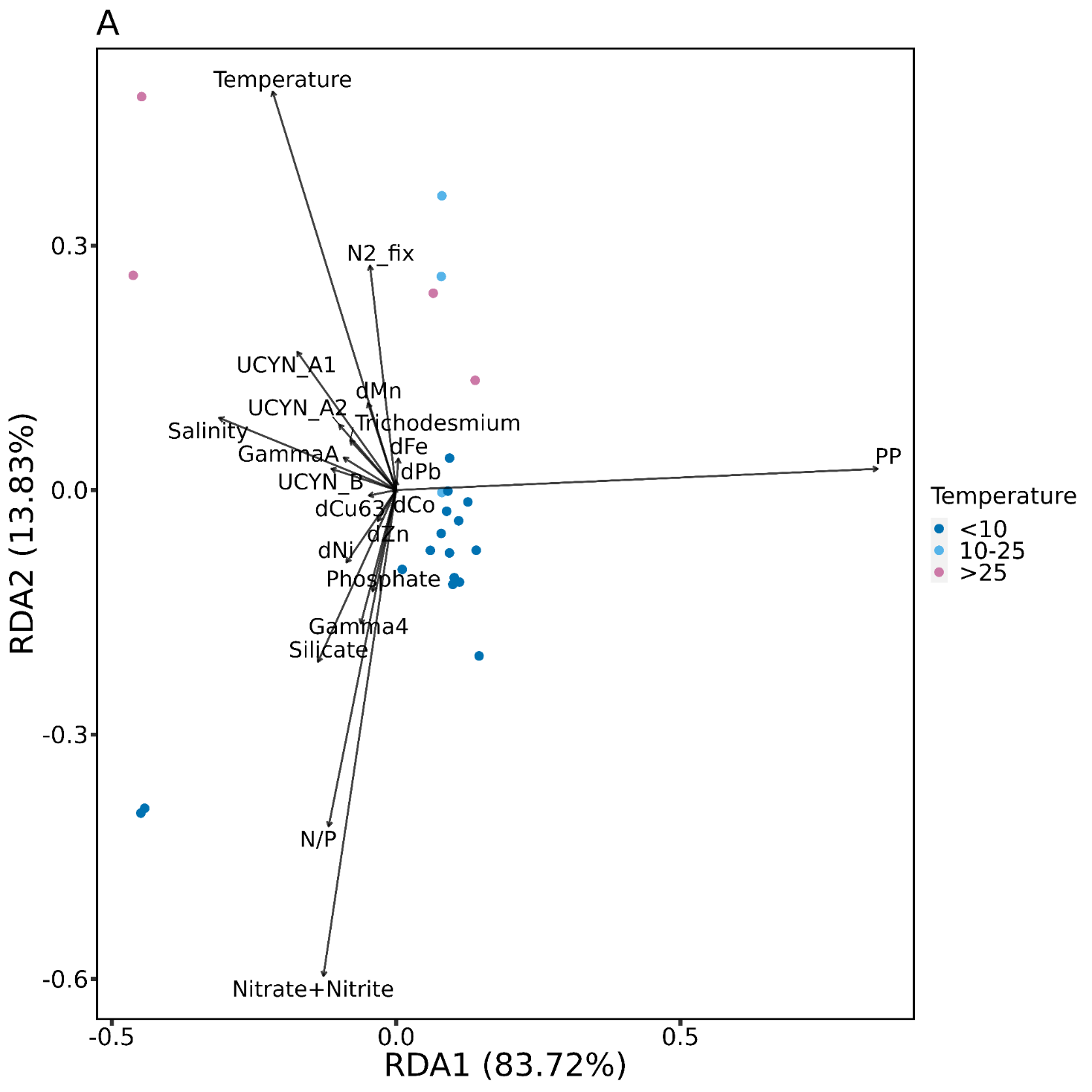


Fig S10: Redundancy analysis (RDA) considering environmental variables and diazotroph abundance based on qPCR *nif*H gene counts.


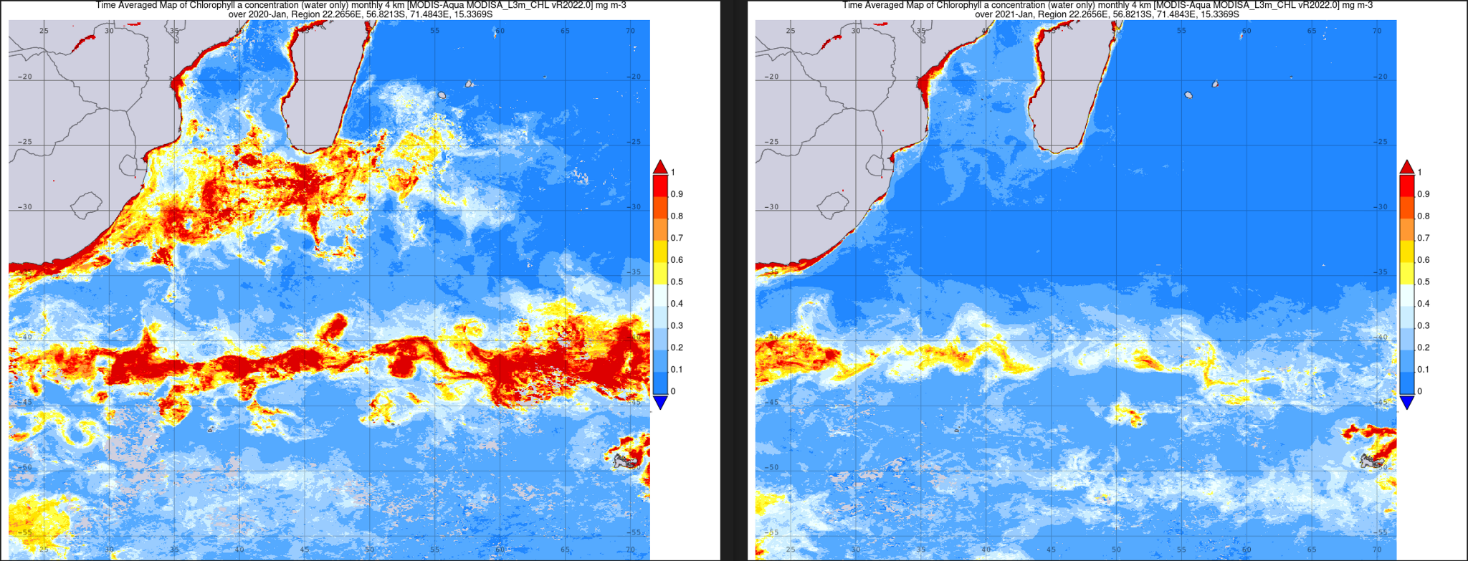


Fig. S11: Comparison of Chlorophyll *a* concentration in January 2020 (left) and January 2021 (right) from Aqua MODIS Satellite Data (L3M 4km product).


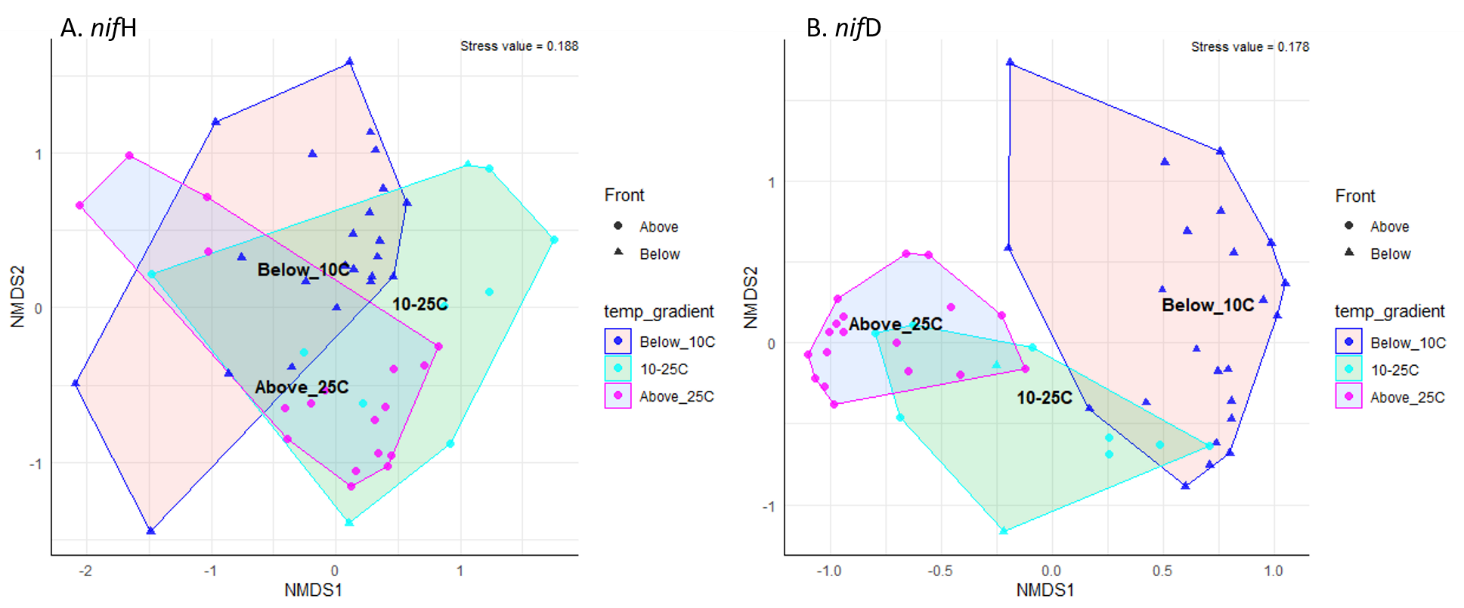


Fig S12: Non-metric multidimensional scaling analysis (NMDS) of (A) *nif*H and (B) *nif*D sequence data across three temperature clusters: below 10°C, 10-25°C, and Above 25°C.


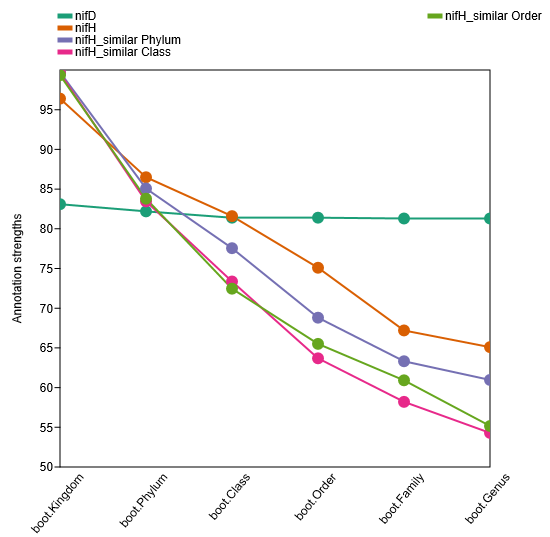


Fig S13: Strength of taxonomic annotations of the top 100 *nif*H and *nif*D ASVs annotated through various databases [*nif*D database (Furbo Reeder, Moynihan and Chowdhury 2023), *nif*H database (Moynihan and Reeder 2023), filtered *nif*H databases at the phylum, class, and order level matched with *nif*D database).

**Supplemental References**

Berthelot H, Duhamel S, L’Helguen S *et al.* NanoSIMS single cell analyses reveal the contrasting nitrogen sources for small phytoplankton. *ISME J* 2019;13:651–62.

Bonnet S, Berthelot H, Turk-Kubo K *et al.* Diazotroph derived nitrogen supports diatom growth in the South West Pacific: A quantitative study using nanoSIMS. *Limnology and Oceanography* 2016;61:1549–62.

Chien YT, Zinder SH. Cloning, functional organization, transcript studies, and phylogenetic analysis of the complete nitrogenase structural genes (nifHDK2) and associated genes in the archaeon Methanosarcina barkeri 227. J Bacteriol 1996;178:143–8.

Church M, Jenkins B, Karl D *et al.* Vertical distributions of nitrogen-fixing phylotypes at Stn Aloha in the oligotrophic North Pacific Ocean. *Aquat Microb Ecol* 2005;38:3–14.

Frank IE, Turk-Kubo KA, Zehr JP. Rapid annotation of nifH gene sequences using classification and regression trees facilitates environmental functional gene analysis. Environmental Microbiology Reports 2016;8:905–16.

Gaby JC, Buckley DH. The Use of Degenerate Primers in qPCR Analysis of Functional Genes Can Cause Dramatic Quantification Bias as Revealed by Investigation of nifH Primer Performance. Microb Ecol 2017;74:701–8.

Halm H, Lam P, Ferdelman TG *et al.* Heterotrophic organisms dominate nitrogen fixation in the South Pacific Gyre. *ISME J* 2012;6:1238–49.

Marie D, Brussaard CPD, Thyrhaug R *et al.* Enumeration of Marine Viruses in Culture and Natural Samples by Flow Cytometry. *Applied and Environmental Microbiology* 1999;65:45–52.

McRose DL, Zhang X, Kraepiel AML *et al.* Diversity and Activity of Alternative Nitrogenases in Sequenced Genomes and Coastal Environments. *Frontiers in Microbiology* 2017;8.

Mise K, Masuda Y, Senoo K et al. Undervalued Pseudo- nifH Sequences in Public Databases Distort Metagenomic Insights into Biological Nitrogen Fixers. Tringe SG (ed.). mSphere 2021;6:e00785-21.

Moisander PH, Beinart RA, Voss M *et al.* Diversity and abundance of diazotrophic microorganisms in the South China Sea during intermonsoon. *ISME J* 2008;2:954–67.

Montoya JP, Voss M, Hler PK *et al.* A Simple, High-Precision, High-Sensitivity Tracer Assay for N2 Fixation. *APPL ENVIRON MICROBIOL* 1996;62.

Moynihan MA, Reeder CF. moyn413/nifHdada2: v2.0.5. 2023, DOI: 10.5281/zenodo.7996213.

Furbo Reeder C, Moynihan M, CHOWDHURY S. nifDdada2. 2023, DOI: 10.5281/zenodo.10124357.

Steward GF, Jenkins BD, Ward BB *et al.* Development and Testing of a DNA Macroarray To Assess Nitrogenase (nifH) Gene Diversity. *Applied and Environmental Microbiology* 2004;70:1455–65.

Thompson A, Carter BJ, Turk-Kubo K *et al.* Genetic diversity of the unicellular nitrogen-fixing cyanobacteria UCYN-A and its prymnesiophyte host. *Environ Microbiol* 2014;16:3238–49.

White AE, Granger J, Selden C *et al.* A critical review of the 15N2 tracer method to measure diazotrophic production in pelagic ecosystems. *Limnology and Oceanography: Methods* 2020;18:129–47.
