# Supplementary material for: Fronts divide diazotroph communities in the Southern Indian Ocean
Source: FEMS Microbiol Ecol. 2024 Jul 11;100(8):fiae095. doi: 10.1093/femsec/fiae095 (PMC11245648; doi:10.1093/femsec/fiae095)
Supplement: fiae095_Supplemental_Files [file fiae095_supplemental_files.zip › supp data FigS6.pdf]

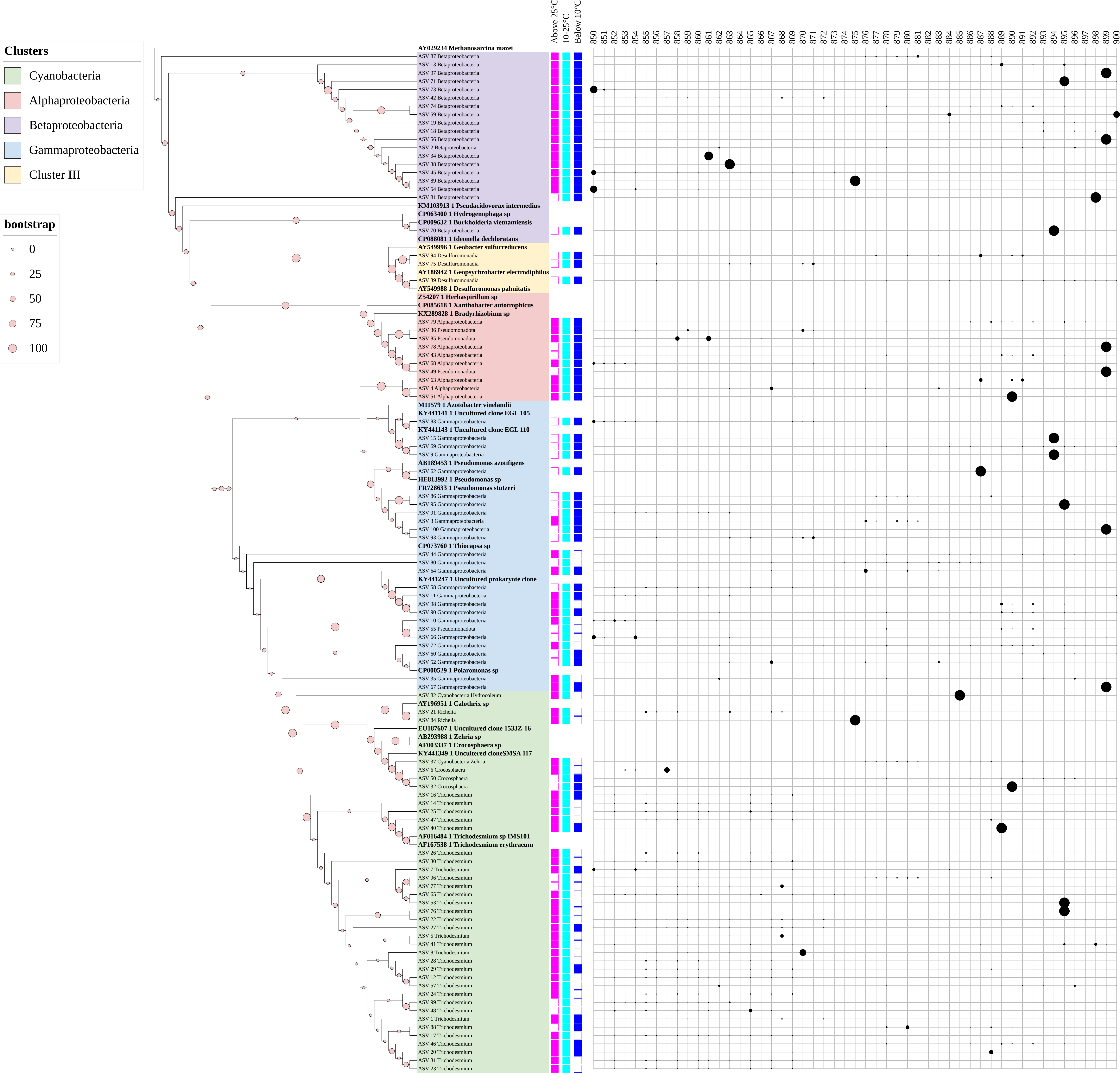

Clusters
Cyanobacteria
Alphaproteobacteria
Betaproteobacteria
Gammaproteobacteria
Cluster III
bootstrap
0
25
50
75
100
Above 25°C
10-25°C
Below 10°C
850 851 852 853 854 855 856 857 858 859 860 861 862 863 864 865 866 867 868 869 870 871 872 873 874 875 876 877 878 879 880 881 882 883 884 885 886 887 888 889 890 891 892 893 894 895 896 897 898 899 900
AY029234 Methanosarcina mazei
ASV 87 Betaproteobacteria
ASV 13 Betaproteobacteria
ASV 97 Betaproteobacteria
ASV 71 Betaproteobacteria
ASV 73 Betaproteobacteria
ASV 42 Betaproteobacteria
ASV 74 Betaproteobacteria
ASV 59 Betaproteobacteria
ASV 19 Betaproteobacteria
ASV 18 Betaproteobacteria
ASV 56 Betaproteobacteria
ASV 2 Betaproteobacteria
ASV 34 Betaproteobacteria
ASV 38 Betaproteobacteria
ASV 45 Betaproteobacteria
ASV 89 Betaproteobacteria
ASV 54 Betaproteobacteria
ASV 81 Betaproteobacteria
KM103913 1 Pseudacidovorax intermedius
CP063400 1 Hydrogenophaga sp
CP009632 1 Burkholderia vietnamiensis
ASV 70 Betaproteobacteria
CP088081 1 Ideonella dechloratans
AY549996 1 Geobacter sulfurreducens
ASV 94 Desulfuromonadia
ASV 75 Desulfuromonadia
AY186942 1 Geopsychrobacter electrodiphilus
ASV 39 Desulfuromonadia
AY549988 1 Desulfuromonas palmitatis
Z54207 1 Herbaspirillum sp
CP085618 1 Xanthobacter autotrophicus
KX289828 1 Bradyrhizobium sp
ASV 79 Alphaproteobacteria
ASV 36 Pseudomonadota
ASV 85 Pseudomonadota
ASV 78 Alphaproteobacteria
ASV 43 Alphaproteobacteria
ASV 68 Alphaproteobacteria
ASV 49 Pseudomonadota
ASV 63 Alphaproteobacteria
ASV 4 Alphaproteobacteria
ASV 51 Alphaproteobacteria
M11579 1 Azotobacter vinelandii
KY441141 1 Uncultured clone EGL 105
ASV 83 Gammaproteobacteria
KY441143 1 Uncultured clone EGL 110
ASV 15 Gammaproteobacteria
ASV 69 Gammaproteobacteria
ASV 9 Gammaproteobacteria
AB189453 1 Pseudomonas azotifigens
ASV 62 Gammaproteobacteria
HE813992 1 Pseudomonas sp
FR728633 1 Pseudomonas stutzeri
ASV 86 Gammaproteobacteria
ASV 95 Gammaproteobacteria
ASV 91 Gammaproteobacteria
ASV 3 Gammaproteobacteria
ASV 100 Gammaproteobacteria
ASV 93 Gammaproteobacteria
CP073760 1 Thiocapsa sp
ASV 44 Gammaproteobacteria
ASV 80 Gammaproteobacteria
ASV 64 Gammaproteobacteria
KY441247 1 Uncultured prokaryote clone
ASV 58 Gammaproteobacteria
ASV 11 Gammaproteobacteria
ASV 98 Gammaproteobacteria
ASV 90 Gammaproteobacteria
ASV 10 Gammaproteobacteria
ASV 55 Pseudomonadota
ASV 66 Gammaproteobacteria
ASV 72 Gammaproteobacteria
ASV 60 Gammaproteobacteria
ASV 52 Gammaproteobacteria
CP000529 1 Polaromonas sp
ASV 35 Gammaproteobacteria
ASV 67 Gammaproteobacteria
ASV 82 Cyanobacteria Hydrocoleum
AY196951 1 Calothrix sp
ASV 21 Richelia
ASV 84 Richelia
EU187607 1 Uncultured clone 1533Z-16
AB293988 1 Zehria sp
AF003337 1 Crocosphaera sp
KY441349 1 Uncultered cloneSMSA 117
ASV 37 Cyanobacteria Zehria
ASV 6 Crocosphaera
ASV 50 Crocosphaera
ASV 32 Crocosphaera
ASV 16 Trichodesmium
ASV 14 Trichodesmium
ASV 25 Trichodesmium
ASV 47 Trichodesmium
ASV 40 Trichodesmium
AF016484 1 Trichodesmium sp IMS101
AF167538 1 Trichodesmium erythraeum
ASV 26 Trichodesmium
ASV 30 Trichodesmium
ASV 7 Trichodesmium
ASV 96 Trichodesmium
ASV 77 Trichodesmium
ASV 65 Trichodesmium
ASV 53 Trichodesmium
ASV 76 Trichodesmium
ASV 22 Trichodesmium
ASV 27 Trichodesmium
ASV 5 Trichodesmium
ASV 41 Trichodesmium
ASV 8 Trichodesmium
ASV 28 Trichodesmium
ASV 29 Trichodesmium
ASV 12 Trichodesmium
ASV 57 Trichodesmium
ASV 24 Trichodesmium
ASV 99 Trichodesmium
ASV 48 Trichodesmium
ASV 1 Trichodesmium
ASV 88 Trichodesmium
ASV 17 Trichodesmium
ASV 46 Trichodesmium
ASV 20 Trichodesmium
ASV 31 Trichodesmium
ASV 23 Trichodesmium
