# Supplementary material for: Fronts divide diazotroph communities in the Southern Indian Ocean
Source: FEMS Microbiol Ecol. 2024 Jul 11;100(8):fiae095. doi: 10.1093/femsec/fiae095 (PMC11245648; doi:10.1093/femsec/fiae095)
Supplement: fiae095_Supplemental_Files [file fiae095_supplemental_files.zip › supp data FigS7.pdf]

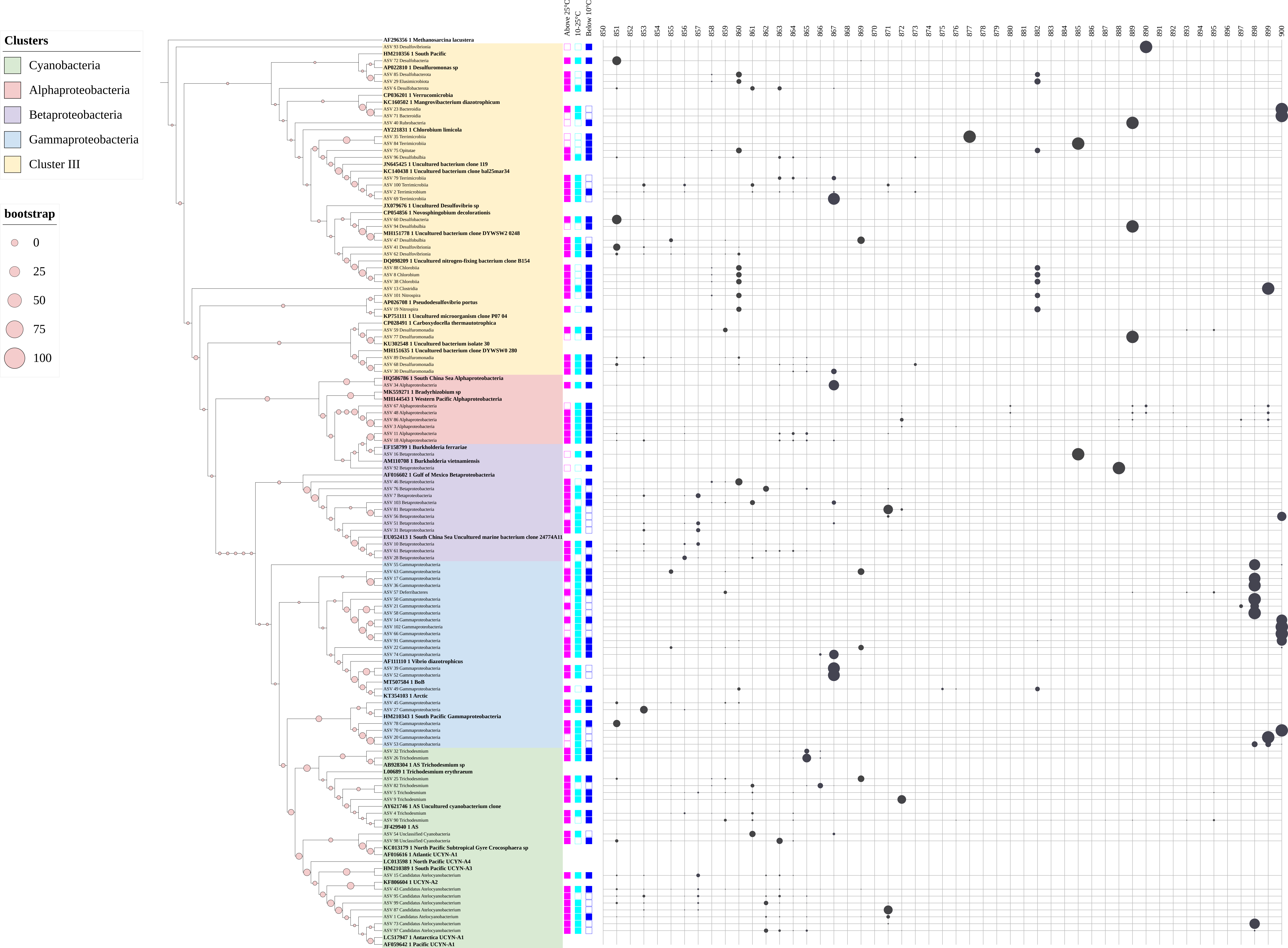

Clusters
Cyanobacteria
Alphaproteobacteria
Betaproteobacteria
Gammaproteobacteria
Cluster III
bootstrap
0
25
50
75
100
Above 25°C
10-25°C
Below 10°C
850 851 852 853 854 855 856 857 858 859 860 861 862 863 864 865 866 867 868 869 870 871 872 873 874 875 876 877 878 879 880 881 882 883 884 885 886 887 888 889 890 891 892 893 894 895 896 897 898 899 900
AF296356 1 Methanosarcina lacustera
ASV 93 Desulfovibrionia
HM210356 1 South Pacific
ASV 72 Desulfobacteria
AP022810 1 Desulfuromonas sp
ASV 85 Desulfobacterota
ASV 29 Elusimicrobiota
ASV 6 Desulfobacterota
CP036201 1 Verrucomicrobia
KC160502 1 Mangrovibacterium diazotrophicum
ASV 23 Bacteroidia
ASV 71 Bacteroidia
ASV 40 Rubrobacteria
AY221831 1 Chlorobium limicola
ASV 35 Terrimicrobiia
ASV 84 Terrimicrobiia
ASV 75 Opitutae
ASV 96 Desulfobulbia
JN645425 1 Uncultured bacterium clone 119
KC140438 1 Uncultured bacterium clone bal25mar34
ASV 79 Terrimicrobiia
ASV 100 Terrimicrobiia
ASV 2 Terrimicrobium
ASV 69 Terrimicrobiia
JX079676 1 Uncultured Desulfovibrio sp
CP054856 1 Novosphingobium decolorationis
ASV 60 Desulfobacteria
ASV 94 Desulfobulbia
MH151778 1 Uncultured bacterium clone DYWSW2 0248
ASV 47 Desulfobulbia
ASV 41 Desulfovibrionia
ASV 62 Desulfovibrionia
DQ098209 1 Uncultured nitrogen-fixing bacterium clone B154
ASV 88 Chlorobiia
ASV 8 Chlorobium
ASV 38 Chlorobiia
ASV 13 Clostridia
ASV 101 Nitrospira
AP026708 1 Pseudodesulfovibrio portus
ASV 19 Nitrospira
KP751111 1 Uncultured microorganism clone P07 04
CP028491 1 Carboxydocella thermautotrophica
ASV 59 Desulfuromonadia
ASV 77 Desulfuromonadia
KU302548 1 Uncultured bacterium isolate 30
MH151635 1 Uncultured bacterium clone DYWSW0 280
ASV 89 Desulfuromonadia
ASV 68 Desulfuromonadia
ASV 30 Desulfuromonadia
HQ586786 1 South China Sea Alphaproteobacteria
ASV 34 Alphaproteobacteria
MK559271 1 Bradyrhizobium sp
MH144543 1 Western Pacific Alphaproteobacteria
ASV 67 Alphaproteobacteria
ASV 48 Alphaproteobacteria
ASV 86 Alphaproteobacteria
ASV 3 Alphaproteobacteria
ASV 11 Alphaproteobacteria
ASV 18 Alphaproteobacteria
EF158799 1 Burkholderia ferrariae
ASV 16 Betaproteobacteria
AM110708 1 Burkholderia vietnamiensis
ASV 92 Betaproteobacteria
AF016602 1 Gulf of Mexico Betaproteobacteria
ASV 46 Betaproteobacteria
ASV 76 Betaproteobacteria
ASV 7 Betaproteobacteria
ASV 103 Betaproteobacteria
ASV 81 Betaproteobacteria
ASV 56 Betaproteobacteria
ASV 51 Betaproteobacteria
ASV 31 Betaproteobacteria
EU052413 1 South China Sea Uncultured marine bacterium clone 24774A11
ASV 10 Betaproteobacteria
ASV 61 Betaproteobacteria
ASV 28 Betaproteobacteria
ASV 55 Gammaproteobacteria
ASV 63 Gammaproteobacteria
ASV 17 Gammaproteobacteria
ASV 36 Gammaproteobacteria
ASV 57 Deferribacteres
ASV 50 Gammaproteobacteria
ASV 21 Gammaproteobacteria
ASV 58 Gammaproteobacteria
ASV 14 Gammaproteobacteria
ASV 102 Gammaproteobacteria
ASV 66 Gammaproteobacteria
ASV 91 Gammaproteobacteria
ASV 22 Gammaproteobacteria
ASV 74 Gammaproteobacteria
AF111110 1 Vibrio diazotrophicus
ASV 39 Gammaproteobacteria
ASV 52 Gammaproteobacteria
MT507584 1 BoB
ASV 49 Gammaproteobacteria
KT354103 1 Arctic
ASV 45 Gammaproteobacteria
ASV 27 Gammaproteobacteria
HM210343 1 South Pacific Gammaproteobacteria
ASV 78 Gammaproteobacteria
ASV 70 Gammaproteobacteria
ASV 20 Gammaproteobacteria
ASV 53 Gammaproteobacteria
ASV 32 Trichodesmium
ASV 26 Trichodesmium
AB928304 1 AS Trichodesmium sp
L00689 1 Trichodesmium erythraeum
ASV 25 Trichodesmium
ASV 82 Trichodesmium
ASV 5 Trichodesmium
ASV 9 Trichodesmium
AY621746 1 AS Uncultured cyanobacterium clone
ASV 4 Trichodesmium
ASV 90 Trichodesmium
JF429940 1 AS
ASV 54 Unclassified Cyanobacteria
ASV 98 Unclassified Cyanobacteria
KC013179 1 North Pacific Subtropical Gyre Crocosphaera sp
AF016616 1 Atlantic UCYN-A1
LC013598 1 North Pacific UCYN-A4
HM210389 1 South Pacific UCYN-A3
ASV 15 Candidatus Atelocyanobacterium
KF806604 1 UCYN-A2
ASV 43 Candidatus Atelocyanobacterium
ASV 95 Candidatus Atelocyanobacterium
ASV 99 Candidatus Atelocyanobacterium
ASV 87 Candidatus Atelocyanobacterium
ASV 1 Candidatus Atelocyanobacterium
ASV 73 Candidatus Atelocyanobacterium
ASV 97 Candidatus Atelocyanobacterium
LC517947 1 Antarctica UCYN-A1
AF059642 1 Pacific UCYN-A1
